# Waste-to-Biofuel Conversion: Hydrodeoxygenation of Frying Oil over NiMoS_2_/Al_2_O_3_ for the Production of Renewable Hydrocarbons

**DOI:** 10.1021/acsomega.6c05430

**Published:** 2026-08-13

**Authors:** Luanne Ester Monteiro Ferreira, Vinicius Rossa, Sancler Vasconcelos, Carolina Vieira Viêgas, Gisel Chenard Díaz, Winny Rego Cardoso, Thiago de Melo Lima, Yordanka Reyes Cruz, Donato Alexandre Gomes Aranda

**Affiliations:** † Department of Organic Process, 28110 UFRJ, Federal University of Rio de Janeiro, Rio de Janeiro 21941-909, Brazil; ‡ Department of Chemical Engineering, UFRJ, Federal University of Rio de Janeiro, Rio de Janeiro 21941-598, Brazil; § Department of Inorganic Chemistry, UFF, Fluminense Federal University, Niterói 24020-007, Brazil

## Abstract

Renewable hydrocarbons were produced from soybean oil (SO) and frying oil (FO) through hydrotreatment over a commercial NiMoS_2_/Al_2_O_3_ catalyst. The effects of temperature (350, 360, and 370 °C), hydrogen pressure (50, 60, and 70 bar), and stirring speed (500, 650, and 800 rpm) were investigated through a 2^3^ factorial design in a batch reactor operated for 5 h with 3 wt % catalyst loading. High catalytic activity was observed for both feedstocks, with X_TG_ (triacylglyceride conversions) above 98% under most experimental conditions. The optimum condition (370 °C, 70 bar, and 800 rpm) resulted in hydrocarbon selectivity (S_HC_) above 97% and selectivity toward the green diesel range (C16–C22 *n*-paraffins) (S_GD_) reached 95.12%. Statistical analysis identified temperature as the most significant influential variable, followed by hydrogen pressure, whereas stirring speed showed no significant effect. The fitted models exhibited high predictive capability (R^2^ > 0.96). Catalyst reuse tests maintained conversions above 93%, although a gradual loss of activity possibly associated with coke formation was observed. The results demonstrate that commercial NiMoS_2_/Al_2_O_3_ catalysts are effective for converting both refined and residual oils into S_GD_, highlighting the potential of frying oil as a low-cost feedstock for sustainable fuel production.

## Graphical abstract

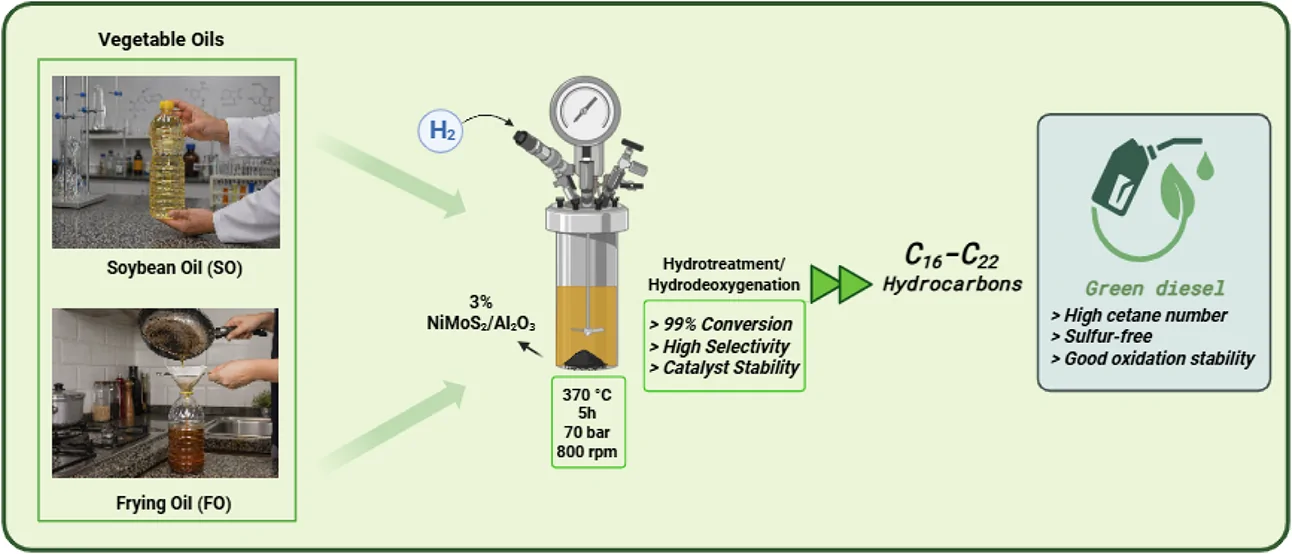

## 1 Introduction

The significant increase in global energy consumption and the intensive use of fossil fuels have intensified worldwide environmental concerns, particularly because of greenhouse gas (GHG) emissions, which contribute to global warming and accelerate climate change. In this context, the production of renewable fuels emerges as a promising alternative, especially when they are of the drop-in type, that is, chemically similar to fossil fuels, ensuring compatibility with existing infrastructure and engines.^1–3^

Hydrotreatment of lipid feedstocks produces oxygen-free paraffinic renewable hydrocarbons, predominantly composed of *n*-paraffins in the diesel boiling range. When appropriate fuel properties are achieved, these hydrocarbons may be referred to as green diesel and can potentially be used as drop-in fuels.^4,5^ To obtain a drop-in product, hydrotreating (HDT) processes—including hydrodeoxygenation (HDO), hydrocracking, and hydrodesulfurization (HDS)—are employed, many of which are adapted from well-established refinery technologies.^3,6^ An important advantage is cost reduction, as existing units can process oils and fats with little or no modification.^7^

Oils and fats are mainly composed of triacylglycerides, which are molecules with a high oxygen content. During hydrotreating, these molecules are subjected to high temperatures and H_2_ pressure in the presence of metallic catalysts, which promotes deoxygenation reactions.^8,9^

Initially, triacylglycerides are cleaved into free fatty acids (R–COOH) and glycerol via hydrogenolysis or hydrogen-assisted hydrolysis. Subsequently, the fatty acids undergo deoxygenation reactions, yielding linear hydrocarbons within the diesel boiling range through pathways such as decarbonylation, decarboxylation, and hydrodeoxygenation (Scheme 1(a)). In parallel, glycerol is hydrogenated and deoxygenated, resulting primarily in propane (Scheme 1(b)).^2,3,10–13^

**Scheme 1:**
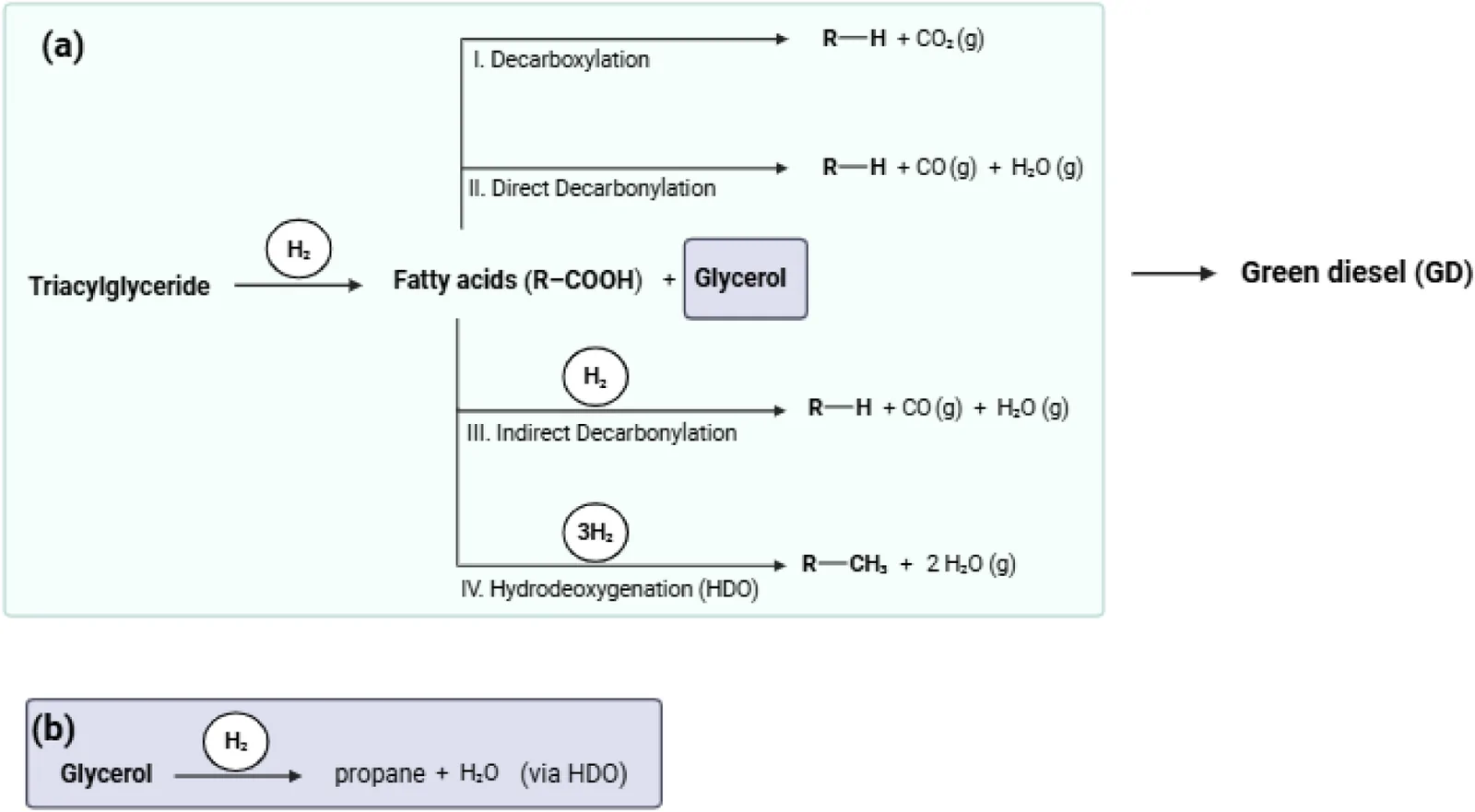
Reaction Pathways for the Hydroprocessing of Triglycerides to Renewable Hydrocarbons in the Diesel Boiling Range: (a) Fatty Acids Undergo Deoxygenation via Decarboxylation, Decarbonylation, and HDO to Form *n*-paraffins, While (b) Glycerol Is Converted into Propane^a^

a R represents alkyl chains derived from fatty acids.

In the direct decarboxylation and decarbonylation reactions (I and II), the carboxyl group is removed from the fatty acid, producing paraffinic hydrocarbons with one fewer carbon atom and releasing oxygen in the form of CO_2_, CO, and H_2_O, respectively. In the indirect decarbonylation route (III), oxygen removal occurs in the form of CO and H_2_O, also resulting in hydrocarbons with one fewer carbon atom. In contrast, in hydrodeoxygenation (IV), oxygen is removed exclusively as water, producing paraffinic hydrocarbons with the same number of carbon atoms as those of the original chain (C_n_).^1,10,14,15^

Alternative technologies have been employed for the development of second-generation fuels, aiming at cost reduction, lower environmental impacts, higher efficiency, and the use of feedstocks that do not compete with the food industry.^16^ In this context, several sources can be utilized, including edible oils, nonedible oils, and FO, among other residual lipid sources. FO refers to fats and oils discarded after use in food processing, cooking, or frying, such as vegetable- and animal-derived oils or fats (soybean oil, corn oil, sunflower oil, lard, chicken fat, etc.).^17–19^

After repeated use, fats and oils become unsuitable for consumption due to oxidation reactions, increased free fatty acid content, and the accumulation of solid residues. However, improper disposal may lead to issues, such as oxygen depletion in water bodies, unpleasant odors in the air, and pipe clogging. Furthermore, the mere degradation of these substances into harmless compounds at high cost is both environmentally and economically inefficient.^14,15^

According to the 2023 report by the Clean Fuels Alliance America (LMC/Global Data), the global market for biofuels derived from FO has grown significantly, reaching a global supply of 3.7 billion gallons, with projections ranging from 5 to 10 billion gallons by 2030. Asia stands out as the main global supplier, with China leading exports since 2017.^18–21^ In 2023, the United States imported approximately 1.4 million metric tons of FO from countries such as China, Canada, Australia, New Zealand, and Chile, representing about US$ 1.66 billion. According to the report, in Latin America, Brazil is the largest exporter of FO, and in 2024, the Ministry of Agriculture and Livestock (MAPA) obtained approval from the United States for FO exports under the International Phytosanitary Certificate (CSIV).^20,21^

It is important to note that when FO is used for biofuel production, it must meet specific standards related to acidity, moisture content, iodine value, among other parameters. However, pretreatment steps—generally aimed at removing suspended solids, water, and free fatty acids from the oils—are influenced by storage conditions. Food residues, nonhermetic containers, and high ambient temperatures may promote decomposition reactions in FO, leading to the formation of polymerization and oxidation products, as well as changes in yield after prolonged periods of improper storage. In addition, the additional costs associated with collection and pretreatment can be significant. Nevertheless, the effects of feedstock degradation occurring between collection, storage, and catalytic conversion remain insufficiently understood.^22,23^

This study aims to evaluate the potential of frying oil as a feedstock for renewable hydrocarbon production through hydrodeoxygenation. Although sulfided NiMo catalysts are well-established in petroleum hydrotreating,^24–27^ they are typically employed to process refinery streams containing relatively low concentrations of oxygenated compounds. In contrast, vegetable oils consist almost entirely of oxygenated triglycerides and free fatty acids, representing a substantially more challenging feedstock. Therefore, a key novelty of the present work is demonstrating that a commercial refinery NiMoS_2_/Al_2_O_3_ catalyst can efficiently convert feedstocks containing nearly 100 wt % oxygenated compounds into green diesel-range hydrocarbons without catalyst modification. This industrially relevant assessment is further strengthened by a systematic comparison between soybean oil and frying oil under identical operating conditions, the application of a statistically designed factorial approach, and the catalyst reuse evaluation.

This contribution is supported by four complementary elements: (i) a rigorous side-by-side comparison of SO and FO under identical reaction conditions, enabling direct assessment of the effect of feedstock quality on catalytic performance; (ii) evaluation of the effects of temperature, hydrogen pressure, and stirring speed through a statistically designed factorial approach on triglyceride conversion (X_TG_), hydrocarbon selectivity (S_HC_), and green diesel selectivity (S_GD_); (iii) the use of a commercially available NiMoS_2_/γ-Al_2_O_3_ catalyst supplied by CENPES/PETROBRAS, widely employed in Brazilian refineries, demonstrating its applicability to biobased feedstocks without catalyst modification; and (iv) catalyst reuse and deactivation studies supported by postreaction characterization. Together, these aspects provide new insights into the use of an industrial hydrotreating catalyst for the production of renewable hydrocarbons from waste-derived feedstocks.^3^

## 2 Materials and Methods

### 2.1 Feedstock and Catalyst

The feedstocks used were commercial refined soybean oil (SO), purchased from a supermarket, and waste frying oil (FO) obtained after domestic frying operations. Both feedstocks were of Brazilian origin. Hydrogen gas (H_2_, 99.9% purity) was supplied by Air Products Brasil. The commercial NiMoS_2_/Al_2_O_3_ catalyst was provided by the Leopoldo Américo Miguez de Mello Research, Development, and Innovation Center (CENPES/PETROBRAS) and is widely used in Brazil in petroleum hydrotreatment processes.^3^

#### 2.1.1 Characterization of SO and FO

The feedstocks were characterized in terms of their chemical, structural, and thermal properties by thermogravimetric analysis, gas chromatography (GC-FID), and Fourier transform infrared spectroscopy (FTIR).

Thermogravimetric analysis was used to quantify the mass variation of the vegetable oils as a function of temperature increase. This analysis aims to evaluate the thermal stability of the samples, as well as to determine ash and moisture contents. The methodology consisted of weighing approximately 10 mg of sample in an alumina crucible and performing the thermal analysis over the temperature range of 20 to 800 °C, with a heating rate of 10 °C/min and an air flow of 100 mL/min. Thermogravimetric analyses were conducted using a PerkinElmer thermogravimetric analyzer, model Pyris 1 TGA.

The oils were characterized by gas chromatography (GC-FID), following method EM 14105, and the acid value was determined according to ASTM D664. The free fatty acid profiles of SO and FO were determined by gas chromatography according to methodology EM 14103.^3^

The infrared absorption spectrum was obtained using a Shimadzu IRPrestige-21 spectrometer within the wavenumber range of 400 to 4000 cm^–1^. This technique was employed to identify and confirm functional groups, as well as to determine the degree of saturation and unsaturation of the vegetable oils.^3^

#### 2.1.2 Catalyst Characterization

For catalyst characterization, the following analyses were performed: hydrogen temperature-programmed reduction (TPR-H_2_), X-ray fluorescence, X-ray diffraction, and full textural analysis using the BET (Brunauer–Emmett–Teller) method.

The catalyst was analyzed by temperature-programmed reduction (TPR-H_2_) using a Micromeritics Autochem 2920 instrument. Hydrogen consumption was monitored by a thermal conductivity detector (TCD). Reduction profiles were normalized considering the sample mass (80 mg) and the H_2_ signal intensity. The sample was previously weighed and introduced into a quartz reactor. Initially, a pretreatment was carried out at 150 °C, with a heating rate of 10 °C/min, for 1 h under a flow of 30 mL/min of argon (Ar). The TPR-H_2_ reduction of the sample was conducted under a flow of 30 mL/min of a mixture containing 2.5% H_2_/Ar, from room temperature up to 900 °C, with a heating rate of 10 °C/min.^3^

X-ray fluorescence (XRF) was used to determine the chemical composition of the catalysts after synthesis. The powdered sample was analyzed using a Rigaku Primini instrument equipped with a palladium X-ray source. X-ray diffraction (XRD) was employed to analyze crystallographic planes, as well as crystal dimensions and orientations. The instrument used was a Rigaku Miniflex II, equipped with a graphite monochromator and using CuKα radiation (30 kV, 15 mA). The analysis was performed over an angular range from 5° to 90°, with an increment of 0.05° every 2 s between points.^3^

Textural analysis was carried out to determine the specific surface area, pore volume, and pore size of the catalyst. The analyses were conducted using a Micromeritics Tristar 3000 Surface Area and Porosimetry Analyzer. The specific surface area was calculated using the BET (Brunauer–Emmett–Teller) method, while pore diameter and specific volume were determined using the BJH method from the adsorption/desorption isotherm. After weighing, the sample was subjected to thermal drying at 200 °C under vacuum (5 × 10^–3^ Torr) for 24 h, and subsequently cooled to room temperature for reweighing before analysis at −196 °C, thereby obtaining N_2_ sorption isotherms (adsorption/desorption) at different partial pressures of N_2_.^3^

### 2.2 Catalytic Tests

#### 2.2.1 Preliminary Catalytic Tests

The experiments were carried out using SO and FO under four different reaction systems: (i) without catalyst, (ii) with alumina (Al_2_O_3_) as catalyst support, (iii) with catalyst reduced for 2 h, and (iv) with catalyst reduced for 4 h. The reactions were conducted in a high-pressure reactor using 40 g of oil and 3 wt % catalyst relative to the oil mass, under constant stirring at 800 rpm and H_2_ pressure of 70 bar for 5 h. Catalyst reduction was performed prior to the reaction under continuous H_2_ flow (99.9%) at a flow rate of 50 mL/min. The reduction process was conducted at 500 °C, maintaining the reduction time according to the experimental condition: 2 or 4 h. After reduction, the catalyst was immediately transferred to the reactor to avoid reoxidation before the start of the catalytic test. The experimental conditions were selected based on real operating conditions of the hydrodesulfurization (HDS) process employed in petroleum refineries for the removal of heteroatoms (S, N, and O) from aviation kerosene and diesel streams. Catalyst reduction was performed ex situ prior to reaction. Although in situ activation is generally preferred to avoid catalyst exposure to air, immediate transfer of the reduced catalyst to the reactor was adopted to minimize reoxidation effects. This procedural choice and its potential impact on the density of active NiMoS edge sites are acknowledged as a limitation of the present study.^28,29^

All reactions were carried out in a stainless-steel autoclave reactor (Parr Instruments Inc., model 4848B), with a total volume of 300 mL, in a simple batch system, under a maximum operating pressure of 200 bar. The reactor was equipped with temperature and pressure controllers, a stirring system, and an external heating mantle.

#### 2.2.2 Statistical Experimental Design

A 2^3^ factorial experimental design (2 levels, 3 factors) was conducted by varying temperature (350–370 °C), hydrogen pressure (50–70 bar), and stirring rate (500–800 rpm) in order to investigate the influence of independent variables on triacylglyceride conversion and selectivity toward hydrocarbons and green diesel. Additionally, three central points were included for each factor, totaling 11 experiments (Tables 1 and 2). Experimental data were analyzed using Protimiza Experimental Design software (https://experimental-design.protimiza.com.br).

**Table 1:** Matrix of 2^3^ Factorial Experimental Design with 3 Central Points to Investigate the Influence of Independent Variables on Hydrocarbons Selectivity (Dependent Variable)

Reactions	Temperature (°C) X1	P_H2_ (bar) X2	Stirring (rpm) X3
1	–1	–1	–1
2	1	–1	–1
3	–1	1	–1
4	1	1	–1
5	–1	–1	1
6	1	–1	1
7	–1	1	1
8	1	1	1
9	0	0	0
10	0	0	0
11	0	0	0

**Table 2:** Real Values of the Levels Used in the Matrix of the Experimental Design 2^3^ + 3 Central Points

	Levels		
Independent variables	–1	0	1
Temperature (°C), X1	350	360	370
P_H2_ (bar), X2	50	60	70
Stirring (rpm), X3	500	650	800

Thus, the hydroprocessing reactions of SO and FO were carried out for all possible combinations of factor levels obtained from the experimental design.

#### 2.2.3 Catalyst Reuse Study

The methodology employed for the catalyst reuse experiments was adapted from the works of Rossa et al. (2017) and Díaz et al. (2025).^3,30^ The experiments were conducted under the following conditions: 40 g of vegetable oil (SO and FO); 3 wt % of catalyst (NiMoS_2_/Al_2_O_3_) relative to the oil mass; temperature of 370 °C; constant hydrogen pressure of 70 bar; reaction time of 5 h; and magnetic stirring at 800 rpm. Prior to the reaction, the catalyst was activated under a hydrogen flow of 50 mL/min at 500 °C for 4 h. A total of five consecutive reaction cycles were performed. After each cycle, the catalyst was recovered by filtration, thoroughly washed with 50 mL of chloroform, 50 mL of isopropanol, and 50 mL of acetone, and subsequently calcined at 300 °C for 4 h using a heating rate of 10 °C/min. The regenerated catalyst was reused in the subsequent cycle, and fresh catalyst was added only to compensate for catalyst losses during recovery and handling. To compensate for unavoidable mechanical losses during catalyst recovery and handling, a proportional amount of fresh catalyst was added in each cycle to maintain the total catalyst loading constant at 3 wt % relative to the oil mass.

The commercial catalyst was intentionally used in its presulfided form without continuous sulfur cofeeding to simulate the direct application of conventional refinery hydrotreating catalysts to sulfur-free renewable feedstocks. Although progressive sulfur depletion may occur under these conditions, the adopted strategy allows for an assessment of catalyst performance during the processing of sulfur-free renewable feedstocks.

### 2.3 Characterization of Reaction Products

#### 2.3.1 Gas Chromatography-FID

Gas chromatography coupled with a flame ionization detector (GC-FID) was employed to identify and quantify products in the liquid phase, including fatty acid methyl esters, hydrocarbons, fatty alcohols, wax esters, and free fatty acids. This technique was also used to evaluate the triacylglycerol conversion and product selectivity. Triacylglycerol conversion was quantified using an external standard method with modifications based on standard EN 14105. Product selectivity, in turn, was determined by using an external calibration method specifically adapted for this analysis.

The analysis of triacylglycerol conversion was performed using a direct injector and a DB5-HT capillary column (30 m length, 0.32 mm internal diameter, and 0.10 μm film thickness, Agilent), with a stationary phase composed of polydimethylsiloxane (PDMS) modified with 5% phenyl-polysiloxane. Operating conditions included an injection volume of 1 μL, helium (99.95%) as the carrier gas with a linear velocity of 92.3 cm/s, and a temperature program starting at 40 °C (1 min), followed by a heating rate of 15 °C/min up to 230 °C, then 7 °C/min up to 320 °C, and finally 30 °C/min up to 380 °C, held for 20 min. The detector temperature was set at 350 °C, and the total analysis time was 48.52 min.

Component identification was performed by comparing the retention times of sample peaks with those of authentic standards, while quantification was carried out by external calibration. It is noteworthy that only the liquid fraction was analyzed, as the study focuses on its application in the production of renewable hydrocarbons in the green diesel and aviation biokerosene boiling ranges. Gaseous products were not directly analyzed; however, their mass was estimated by difference. Calibration curves for the oil samples were constructed using standard solutions with mass fractions of 0.50, 0.25, 0.10, 0.01, 0.001, and 0.0001. For each concentration, the area corresponding to the triacylglycerol region was determined. Sample preparation for these calibrations involved collecting 11 μL of sample, adding 22 μL of the derivatizing agent MSTFA, and allowing a waiting time of 30 min prior to dilution in 1 mL of a 1:1 hexane/isopropanol solution. Regression equations were then obtained for the triacylglycerol mass fractions in SO and FO. Conversion was calculated based on the initial and final molar fractions of triacylglycerols in each sample, according to Equation 1

1 $$X_{TG}\%=\left(\frac{w_{i-w_{f}}}{w_{i}}\right)\times 100$$

where *X*_TG_% is the triacylglycerol conversion (%); *w_i_* is the molar fraction of triacylglycerols at the beginning of the reaction; and *w_f_* is the molar fraction of triacylglycerols at the end of the reaction.

Selectivity analysis was conducted using a split injector (split ratio 1:25) and the same DB5-HT column described above. Helium (99.95%) was used as the carrier gas with a linear velocity of 38 cm/s. The temperature program started at 50 °C, followed by heating at 4 °C/min up to 180 °C, then 5 °C/min up to 320 °C, and subsequently 30 °C/min up to 385 °C, maintained for 15 min. The detector temperature was set at 260 °C, and the total analysis time was 77.76 min. For the analysis, 13 μL of each sample was diluted in 1 mL of a hexane/isopropanol solution (1:1).

Product selectivity was determined by calculating the relative area of each compound with respect to the total area of all identified products, as defined in Equation 2 This approach enabled the evaluation of product distribution formed during the reaction, providing insights into catalytic behavior and process efficiency.

2 $$S_{i}=\frac{Area_{i}}{Area_{\mathrm{all products}}}\cdot 100$$

where *S_i_* is the selectivity to product *i*; Area_i_ is the peak area of compound *i*; and Area_all products_ is the sum of the peak areas of all formed products.

This methodology was designed to determine the fatty acid composition of vegetable oils. For this purpose, 300 mg of each oil sample was weighed, and the following procedure was applied: initially, a saponification reaction was carried out by reacting the oils with 2 mL of a saturated potassium hydroxide solution (0.5 mol/L) in methanol, followed by an esterification step using 2 mL of a methanolic solution containing 5% hydrochloric acid. The reactions were conducted in a closed system at 75 °C using a water bath for 10 min. Subsequently, to separate the methylated fatty acids from other components, 2 mL of distilled water and 2 mL of analytical-grade hexane were added. The organic phase containing hexane was then transferred to the second stage.

In the second stage, the produced methyl esters were analyzed using a Shimadzu gas chromatograph, model GC-2014, equipped with a flame ionization detector (FID), a split/splitless injector, and a Carbowax 20M capillary column with a polyethylene glycol stationary phase (30 m length, 0.25 mm internal diameter, and 0.25 μm film thickness). Hydrogen was used as the carrier gas with a linear velocity of 26.5 cm/s. The oven temperature program was defined as follows: 60 °C for 2 min; heating from 60 to 200 °C at 10 °C/min; then from 200 to 240 °C at 5 °C/min, with a final hold at 240 °C for 15 min. The injector temperature was maintained at 250 °C, the detector at 280 °C, and the injection volume was 1 μL.

The identification of methyl esters was performed by comparing the retention times of each sample component with those of a mixture of 37 external standards of fatty acid methyl esters (FAMEs), covering the range from C4:0 to C24:0, supplied by Sigma. The composition was determined by area normalization, comparing the relative percentage of each characteristic FAME peak with the total peak area in the chromatogram.

#### 2.3.2 Fourier Transform Infrared Spectroscopy (FTIR)

SO and FO were characterized by Fourier Transform Infrared Spectroscopy (FTIR). Reaction products obtained from preliminary tests were also analyzed by FTIR in order to evaluate the removal of oxygenated functional groups from the molecules present in SO and FO. The analyses were carried out using a Shimadzu IR PRESTIGE-21 instrument. Spectra were recorded in the range of 4000 to 400 cm^–1^, with a resolution of 1 cm^–1^ and 64 scans per spectrum.

## 3 Results and Discussion

### 3.1 Feedstock Characterization

Initially, the feedstocks (SO and FO) were characterized by thermal analysis in order to evaluate the thermal stability of the oils as well as their moisture and ash contents.

Figure 1 presents the thermogravimetric analysis curves of the vegetable oils SO and FO, while Table 3 summarizes the main thermal events and mass loss (%), in addition to the moisture (%) and ash (%) contents of the vegetable oils.

**Figure 1:**
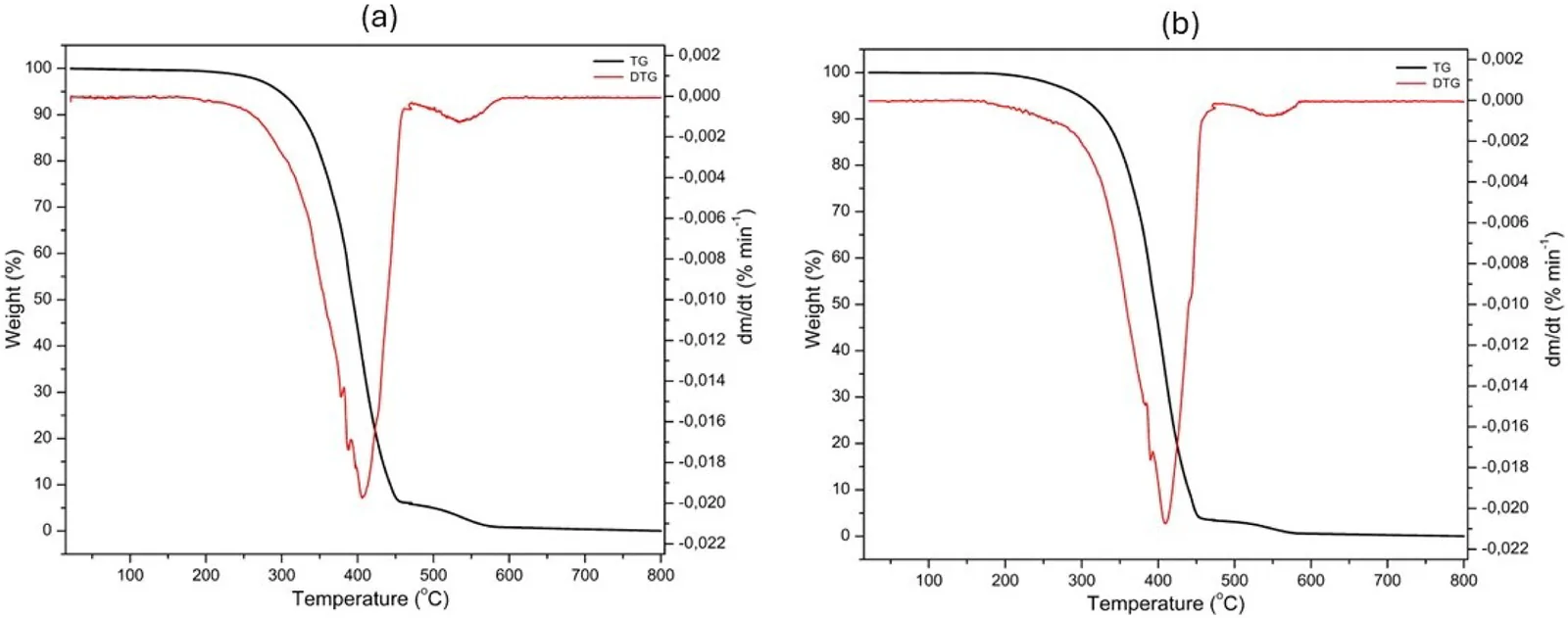
Thermogravimetric analysis (TG and DTG) of (a) soybean oil: SO; and (b) frying oil: FO.

**Table 3:** Main Thermal Events and Mass Loss (%), Moisture (%), and Ash (%) for Each of the Oils (SO and FO)

Event	Temperature range (°C)	Assignment	Mass loss (%) SO	Mass loss (%) FO
I	20–200	Loss of physically adsorbed moisture	0.74	0.46
II	201–383	Decomposition of polyunsaturated fatty acid species	68.07	70.85
III	384–391	Decomposition of monounsaturated fatty acid species	11.57	10.42
IV	392–588	Decomposition of saturated fatty acids and heavy residues	13.52	14.58
V	>450	Oxidation of residual species under air atmosphere, accompanied by oxygen uptake and peroxide formation	5.72	3.25
Total loss	-	-	99.62	99.56
Moisture	-	-	0.74	0.46
Ash	800 °C	Residual inorganic content	0.38	0.44

Based on the TG/DTG data presented in Figure 1 and Table 3, and supported by the specialized literature, the thermal events observed for each vegetable oil (SO and FO) were analyzed over the temperature range from 20 to 800 °C. Event I, observed between 20 and 200 °C, is attributed to moisture evaporation and is consistent with the moisture contents determined for SO (0.74%) and FO (0.46%). According to previous studies, the first major thermal decomposition stage (Event II) is mainly associated with the degradation of polyunsaturated fatty acid species, corresponding to the onset of triglyceride decomposition and representing the most important stage for assessing the thermal stability of edible oils. The second decomposition stage (Event III) has been attributed mainly to the thermal degradation of monounsaturated fatty acid species. During this process, cleavage of carbon–carbon double bonds may occur, promoting the formation of more saturated structures. The third decomposition stage (Event IV) is mainly associated with the decomposition of saturated fatty acid species, which exhibit higher thermal stability.^31^

During heating, triacylglycerides, which account for 96–98% of the composition of vegetable oils, generate volatile compounds that are continuously removed by the vapor produced. These products are mainly formed through thermal reactions involving unsaturated fatty acids. The initial stage of oxidation in vegetable oils is characterized by oxygen uptake along the fatty acid chains, resulting in the formation of oxidation products such as peroxides. This behavior can be observed as an apparent increase in sample mass, detected after 450 °C for all analyzed oils, defined as event V. Considering that the analyses were conducted under an oxidizing atmosphere, a slight mass gain was detected in the DTG curves, indicating that the thermal decomposition process involves both absorption and release of the oxidizing gas (compressed air). This phenomenon was observed for both oils in the range between 450 and 600 °C.^31,32^

Finally, the ash content, measured at 800 °C, showed low values: 0.38% for SO and 0.44% for FO. These levels are considered insufficient to cause significant interference in the HDO process, particularly with regard to catalyst deactivation.

Table 4 presents the characterization results of SO and FO in terms of the chemical composition of triacylglycerides (TAGs), diacylglycerides (DAGs), monoacylglycerides (MAGs), and free fatty acids (FFAs), obtained by gas chromatography (GC-FID).

**Table 4:** Results of the Chemical Composition of Vegetable Oils (SO and FO) in Terms of Triacylglycerides (TAG), Diacylglycerides (DAG), Monoacylglycerides (MAG), Free Fatty Acids (FFA), and Acid Index

Oils	TAG (%)	DAG (%)	MAG (%)	FFA (%)	Acid index (mg KOH g^–1^)
SO	98.30	0.56	0.26	0.88	0.01
FO	95.18	3.32	0.01	1.49	0.21

The amounts of TAG, DAG, MAG, and FFA directly influence the amount of H_2_ required for the HDO of vegetable oils, aiming at their conversion into hydrocarbons.^33–38^ The greater the number of oxygenated groups and the degree of unsaturation of carbon–carbon bonds in vegetable oil chains, the higher the amount of H_2_ required to hydrolyze glycerol from TAG, DAG, and MAG; saturate double bonds in the carbon chains; and promote the removal of oxygenated groups from the molecules via hydrodeoxygenation. The acidity index indicates the amount of free FFA; therefore, the higher this index, the greater the concentration of these free fatty acids in vegetable oils. The free FFA profile was also determined by gas chromatography coupled with a flame ionization detector (GC-FID), as presented in Table 5.

**Table 5:** Profile of Vegetable Oils (SO and FO) in Terms of Free Fatty Acids (FFA)

	Fatty acids (%)	
Composition	SO	FO
Lauric acid (C12:0)	-	0.04
Myristic acid (C14:0)	-	0.01
Pentadecanoic acid (C15:0)	0.05	0.13
Palmitoleic acid (C16:1)	10.20	12.47
Heptadecanoic acid (C17:1)	0.08	0.10
Estearic acid (C18:0)	0.07	0.07
Oleic acid (C18:1)	32.68	32.73
Linoleic acid (C18:2)	50.50	48.40
Linolenic acid (C18:3)	5.42	5.17
Nanodecanoic acid (C19:0)	0.14	0.03
Eicosanoic acid (C20:3)	0.03	0.47
Eicosapentaenoic acid (C20:5)	0.26	0.22
Erucic acid (C22:1)	-	-
Docosadienoic acid (C22:2)	0.05	-
Tricosanoic acid (C23:0)	0.54	-
Lignoceric acid (C24:0)	-	0.15
Saturated	0.79	0.43
Unsaturated	99.21	99.56
Monounsaturated	42.96	45.30
Dienounsaturated	50.55	48.40
Trienounsaturated	5.45	5.64
Polyunsaturated	0.25	0.22

In addition to the quantified glyceride and free fatty acid fractions, vegetable oils may naturally contain minor constituents such as sterols, tocopherols, phospholipids, pigments, and other unsaponifiable compounds.^39,40^ However, the soybean oil (SO) employed in this study was a commercially refined edible oil. During industrial refining processes—including degumming, neutralization, bleaching, and deodorization—most of these minor constituents are removed, remaining only at trace levels that are generally considered negligible for hydroprocessing reactions. Consequently, the chemical composition of the refined SO is adequately represented by the quantified TAG, DAG, MAG, and FFA fractions.

The frying oil (FO) used in this work was originally the same refined soybean oil and, therefore, had also undergone the same refining process. Nevertheless, repeated exposure to high temperatures during frying promotes oxidation, hydrolysis, and polymerization reactions, leading to the formation of small amounts of secondary oxidation products, including hydroperoxides, aldehydes, ketones, and other oxygenated species. Although these compounds were not quantified separately because of their very low concentrations, they may also undergo hydrodeoxygenation and secondary hydrocracking under the reaction conditions employed, contributing to the formation of lighter hydrocarbons.^41^ The absence of additional significant FTIR bands and the thermogravimetric behavior of both feedstocks indicate that these species, if present, occur only in minor amounts and, therefore, are not expected to significantly affect the overall interpretation of the catalytic results.

On the basis of Table 5, a qualitative estimation of hydrogen demand can be inferred from the degree of unsaturation of the fatty acid profiles, suggesting a slightly higher hydrogen demand for SO compared to FO.

The HDT route via HDO of oils and fats, with the addition of H_2_ from both fossil and renewable sources, is a widely studied technology for converting triacylglycerides, diacylglycerides, monoacylglycerides, and their respective fatty acids to hydrocarbons. This process enables the production of green paraffinic hydrocarbons, with carbon chain lengths equal to or one carbon unit shorter than those of the original fatty acid.^16,33,35,36,38,42–46^

Figure 2 shows the infrared spectra of SO and frying oil FO. Table 6 lists the assignments of the main bands in the infrared spectra according to the reference literature.^47,48^

**Figure 2:**
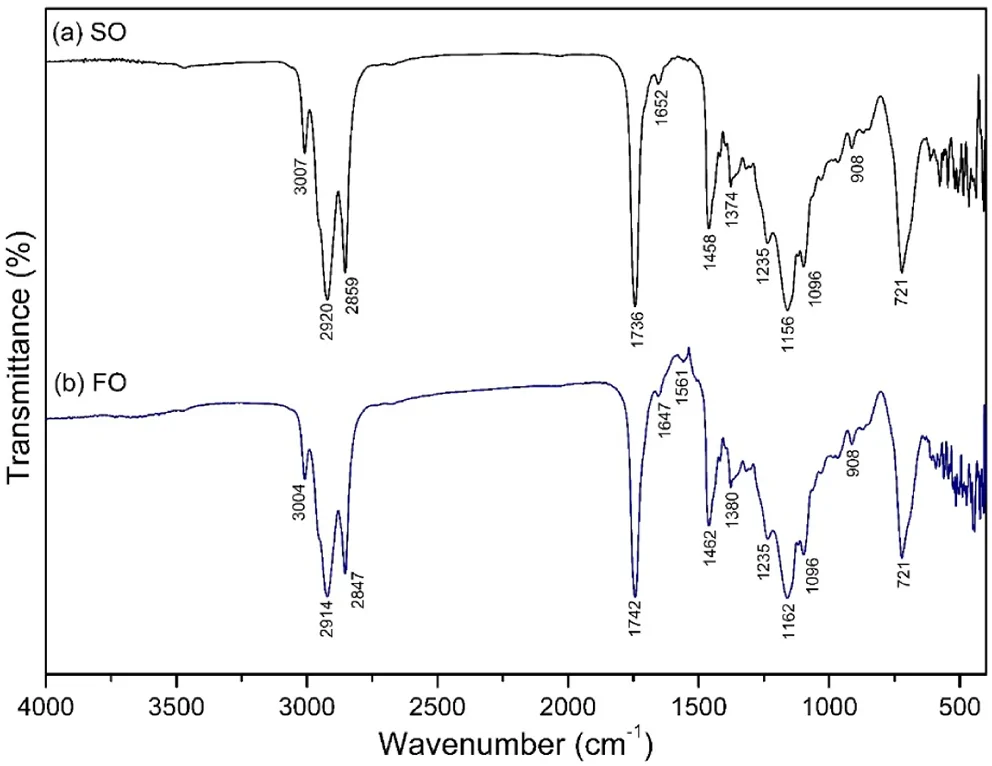
Infrared spectra of soybean oil (SO) and frying oil (FO).

**Table 6:** Assignments of the Main Bands in the Infrared Spectra for the Raw Materials Soybean Oil (SO) and Frying Oil (FO)

Wavenumbers (cm^–1^)		
SO	FO	Assignments of the main bands
3007	3004	Deformation of the bonds in the =CH group
2920	2914	Stretching of the bonds present in the −CH_2_ and −CH_3_ groups
2859	2847	Stretching of the bonds present in the −CH_2_ and −CH_3_ groups
1736	1742	Vibration of the bonds in the —C=O esters group
1652	1647	Axial deformation of the C=C bond
-	1561	Stretching of the bonds present in the −CH_2_ and −CH_3_ groups
1458	1462	Angular deformation in the bonds of the CH_2_ group
1374	1380	Angular deformation in the bonds of the CH_3_ group
1235	1235	Vibration of the bonds in the −C–O esters group
1156	1162	Vibration of the bonds in the −C–O esters group
1096	1096	Vibration of the bonds in the −C–O–C– group
908	908	Out-of-plane angular deformation of the —RCH=CH_2_ bonds
721	721	Stretching of the bonds present in the −CH_2_ and −CH_3_ groups

The regions characteristic of triacylglycerides are identified by bands at 1740 cm^–1^, attributed to —C=O ester bond vibrations, and in the range of 1235–1096 cm^–1^, corresponding to −C–O– and −C–O–C– bond vibrations, typical of esters. These bands are assigned to triacylglycerides and their precursor esters linked to the glycerol molecule.

### 3.2 Catalyst Characterization

Figure 3 presents the temperature-programmed reduction profile (TPR-H_2_) of the commercial NiMoS_2_/Al_2_O_3_ catalyst.

**Figure 3:**
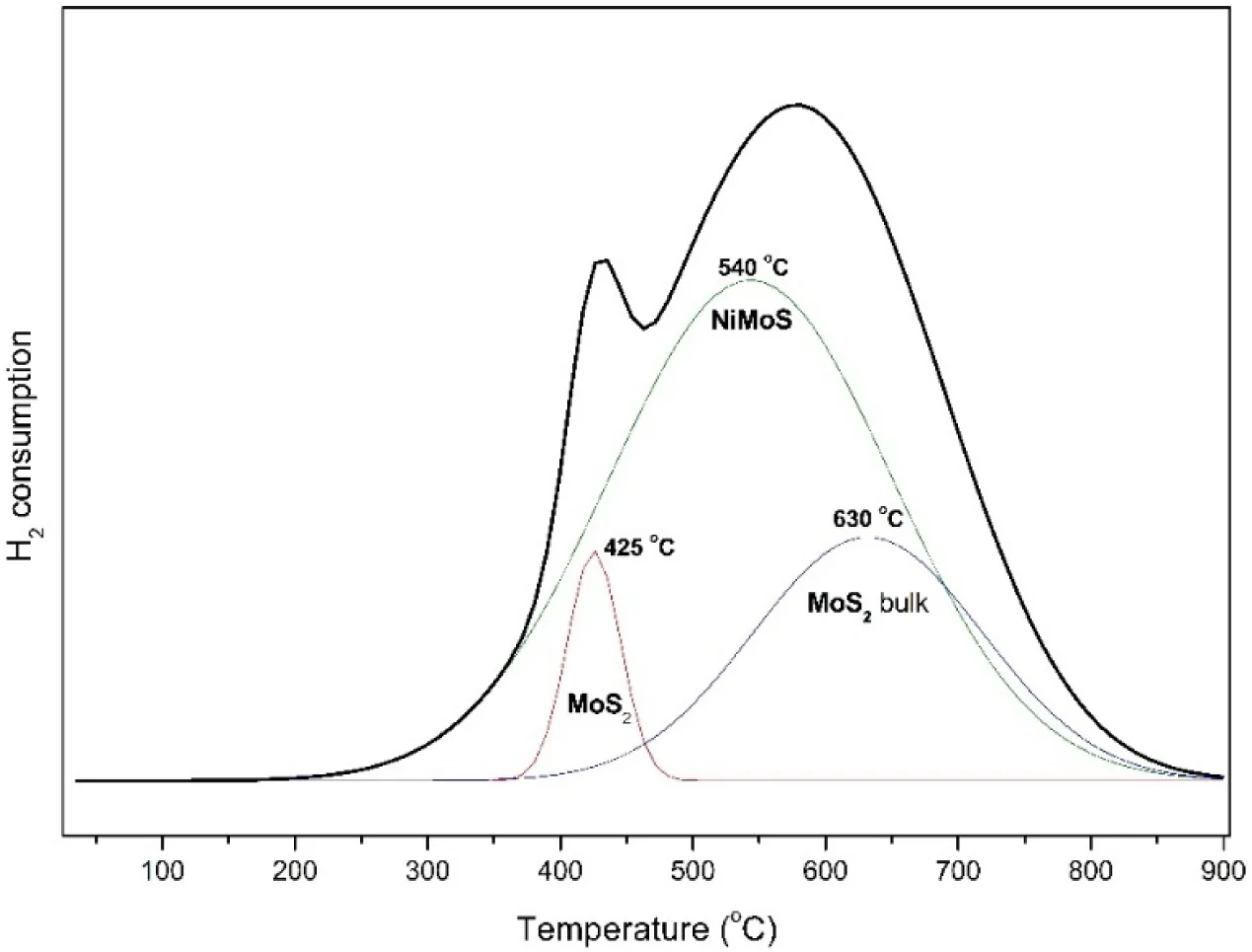
TPR-H_2_ profile of the commercial NiMoS_2_/Al_2_O_3_ catalyst and its deconvolution according to Díaz et al. (2024).

The TPR profile of the commercial NiMoS_2_/Al_2_O_3_ catalyst exhibited three peaks after deconvolution, similar to those reported by Rodríguez-Catellón (2008). Figure 3 shows a peak at 426 °C associated with the reduction of weakly adsorbed sulfur on the support surface, representing the reduction of sulfur vacancies with the formation of the MoS_2_ phase. A second peak, at 542 °C, was attributed to the reduction of nickel sulfide species located at the edges of MoS_2_ layers, corresponding to the NiMoS phase, which is recognized for its high catalytic activity.^49^ The third peak, at 631 °C, is likely associated with the reduction of basal planes, indicative of bulk MoS_2_.^49–52^ This H_2_-TPR analysis indicates the presence of sulfur/sulfide species in the catalyst both before and after hydrogen reduction.

The qualitative and quantitative compositions of the commercial NiMoS_2_/Al_2_O_3_ catalyst and the NiMoS_2_/Al_2_O_3_ catalyst after 4 h of reduction followed by passivation were determined by X-ray fluorescence (XRF) analysis, as presented in Table 7.

**Table 7:** Chemical Composition of the Commercial NiMoS_2_/Al_2_O_3_ Catalyst and after the Reduction Process for 4 h (50 mL min^–1^ H_2_ Flow for 4 h) and Passivation, Obtained by X-ray Fluorescence (XRF) According to Díaz et al. (2024)

	Chemical composition (%, m/m)			
Catalyst	Al_2_O_3_	MoO_3_	NiO	SO_3_
NiMoS_2_/Al_2_O_3 commercial_	55.33	34.90	7.46	2.29
NiMoS_2_/Al_2_O_3 reduced 4 h_	55.35	34.92	7.46	2.29

The qualitative and quantitative XRF analysis, presented in Table 7, confirmed the presence of Ni, Mo, and S derived from the catalyst precursors, as well as Al from the support. No significant variation in bulk sulfur content was observed after reduction and passivation. This behavior was expected and indicates that no appreciable loss of the main active components occurred during these treatments, supporting the effectiveness of the passivation procedure and the reliability of the characterization protocol employed. The atomic ratio Ni/(Ni+Mo), which is important for hydrotreatment performance, was approximately 0.20, a value within the effective range (0.20–0.40) for the conversion of triacylglycerides into hydrocarbons via HDO, as reported by Kubička (2010). Studies with alumina-supported NiMo catalysts have reported conversion and selectivity up to 100% at temperatures up to 280 °C, contact times up to 4 h, and hydrogen pressures of 35 bar.^53^

X-ray diffraction (XRD), shown in Figure 4, revealed the diffractograms of the commercial NiMoS_2_/Al_2_O_3_ catalyst and those obtained after reduction for 2 and 4 h.

**Figure 4:**
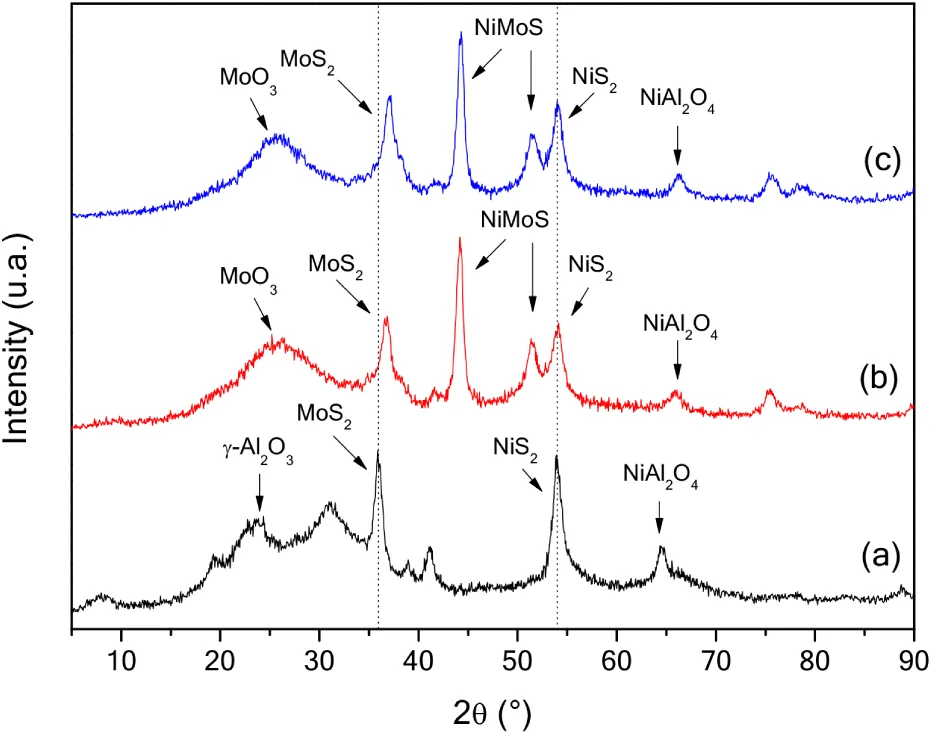
X-ray diffractograms of the catalyst (a) commercial NiMoS_2_/Al_2_O_3_, (b) NiMoS_2_/Al_2_O_3_ reduced for 2 h, and (c) NiMoS_2_/Al_2_O_3_ reduced for 4 h.

Figure 4(a) presents the characteristic X-ray diffraction (XRD) pattern of the commercial NiMoS_2_/Al_2_O_3_ catalyst.^54^ Figure 4(b) and 4(c) show the diffraction patterns of the NiMoS_2_/Al_2_O_3_ catalyst after reduction for 2 and 4 h, respectively. A low-intensity peak associated with the monoclinic Mo_3_O phase was observed at 2θ ≈ 27°, while the MoS_2_ phase exhibited a diffraction peak at 2θ ≈ 36°. Peaks located at 2θ ≈ 34° and 50° are attributed to the NiMoS phase, and a diffraction signal corresponding to the NiS_2_ phase was detected at 2θ ≈ 54°.^54–60^ The presence of these sulfur-containing phases indicates the incorporation of sulfide groups in the commercial NiMoS_2_/Al_2_O_3_ catalyst, as also evidenced by the TPR analysis (Figure 3).

All diffraction peaks observed for this catalytic material are in agreement with those reported in the literature, in which the authors identified crystalline phases using reference files from the JCPDS (Joint Committee on Powder Diffraction Standards) database.

The specific surface area (determined by the BET method), total pore volume, and average pore diameter were obtained from N_2_ adsorption–desorption measurements. The corresponding values are summarized in Table 8.

**Table 8:** Textural Characteristics of Al_2_O_3_ Support, Commercial NiMoS_2_/Al_2_O_3_ Catalyst, and NiMoS_2_/Al_2_O_3_ Catalyst Reduced for 4 h^a^

Catalyst	S_BET_ (m^2^ g^–1^)	V_pores_ (cm^3^ g^–1^)	D_pores_ (nm)
Al_2_O_3_	161	0.38	7.8
NiMoS_2_/Al_2_O_3 commercial_	104	0.09	3.3
NiMoS_2_/Al_2_O_3 reduced 4 h_	84	0.27	11.8

a S_BET_: specific surface area (m^2^ g^–1^); V_pores_: average pore volume (cm^3^ g^–1^); D_pores_: average pore diameter.

Regarding the textural properties obtained by N_2_ adsorption/desorption (Table 8), the Al_2_O_3_ support exhibited a specific surface area of 161 m^2^ g^–1^ and an average pore diameter of 7.8 nm, confirming its mesoporosity. After metal incorporation, the average pore diameter decreased to 3.3 nm without loss of mesoporosity, which is suitable for reactions involving large molecules such as triglycerides, as it improves diffusion pathways. For the NiMoS_2_/Al_2_O_3_ catalyst, the surface area decreased from 104 to 84 m^2^ g^–1^ after 4 h of reduction; however, an increase in pore volume and average pore diameter was observed, which may enhance accessibility to active sites and improve interaction with sterically demanding substrates. These results are consistent with previous literature reports, including the study by Łaniecki et al. (2000).^61,62^

### 3.3 Hydrodeoxygenation of Vegetable Oils via H_2_ and Catalytic Activity

The hydrodeoxygenation reactions were carried out using SO and FO in order to evaluate the influence of the commercial NiMoS_2_/Al_2_O_3_ catalyst on hydrocarbon selectivity. The evaluation was compared with control reactions without a catalyst and with the use of the support material alone. The impact of catalyst reduction time (activation for 2 and 4 h) on maximizing hydrocarbon selectivity was also investigated.

The catalytic tests were conducted under the following conditions: 40 g of vegetable oil (SO or FO), 3 wt % of NiMoS_2_/Al_2_O_3_ catalyst relative to the oil mass, external hydrogen pressure of 70 bar, temperature of 370 °C, stirring at 800 rpm, for 5 h. For comparison, four different reactions were carried out with each oil: a control reaction without catalyst (RS0 for SO and RF0 for FO); a reaction using only the Al_2_O_3_ support (RS1/RF1); a reaction with NiMoS_2_/Al_2_O_3_ catalyst activated by reduction at 500 °C under H_2_ flow (99.9%) of 50 mL min^–1^ for 2 h (RS2h/RF2h); and a similar reaction with reduction for 4 h (RS4h/RF4h). The complete results are described in Tables 9 and 10.

**Table 9:** Catalytic Tests Using Soybean Oil (SO)^a^

	Reactions			
Conditions	RS0	RS1	RS2h	RS4h
P_H2_ (bar)	70.00	70.00	70.00	70.00
Catalyst	-	Al_2_O_3_	NiMoS_2_/Al_2_O_3_	NiMoS_2_/Al_2_O_3_
Reduction time (h)	-	-	2	4
Glycerol quantity	94.50	96.25	98.35	98.82
SO conversion (%)	97.53	85.09	88.09	78.46
Liquid products (%)	2.47	14.91	11.91	21.54
**Functional groups**	**Selectivity (%)**			
Fatty acids (%)	0.57	1.56	-	0.01
Methyl esters (%)	83.41	80.81	13.84	3.47
Fatty alcohols (%)	-	-	0.86	0.22
Wax (%)	1.04	-	0.29	0.01
Hidrocarbons (%)	14.99	17.63	85.01	96.30

a RS0: control reaction without catalyst; RS1: reaction using only Al_2_O_3_ as support; RS2h: reactions using the NiMoS_2_/Al_2_O_3_ catalyst reduced at 500 °C with H_2_ flow (99.9%) of 50 mL/min for 2 h; RS4h: reactions using the NiMoS_2_/Al_2_O_3_ catalyst reduced at 500 °C with H_2_ flow (99.9%) of 50 mL/min for 4 h.

**Table 10:** Catalytic Tests Using Frying Oil (FO)^a^

	Reactions			
Conditions	RF0	RF1	RF2h	RF4h
P_H2_ (bar)	70.00	70.00	70.00	70.00
Catalyst	-	Al_2_O_3_	NiMoS_2_/Al_2_O_3_	NiMoS_2_/Al_2_O_3_
Reduction time (h)	-	-	2	4
Glycerol quantity	96.26	97.85	98.68	98.98
FO conversion (%)	82.68	96.68	85.93	89.87
Liquid products (%)	17.32	3.32	14.07	10.13
**Functional groups**	**Selectivity (%)**			
Fatty acids (%)	1.76	1.09	0.00	0.00
Methyl esters (%)	78.79	82.12	17.63	1.50
Fatty alcohols (%)	0.00	0.67	2.11	0.21
Wax (%)	0.00	0.00	3.08	0.61
Hidrocarbons (%)	19.45	16.13	77.17	97.68

a RF0: control reaction without catalyst; RF1: reaction using only Al_2_O_3_ as support; RF2h: reactions using the NiMoS_2_/Al_2_O_3_ catalyst reduced at 500 °C with H_2_ flow (99.9%) of 50 mL/min for 2 h; RF4h: reactions using the NiMoS_2_/Al_2_O_3_ catalyst reduced at 500 °C with H_2_ flow (99.9%) of 50 mL/min for 4 h.

The use of the NiMoS_2_/Al_2_O_3_ catalyst reduced for 2 h promotes a significant increase (from ∼15% (SO) and ∼20% (FO) to ∼85% (SO) and ∼77% (FO)) in hydrocarbon selectivity during the HDO process of vegetable oils. Extending the reduction time to 4 h results in a further increase in selectivity, demonstrating the fundamental importance of the active metallic phase of the catalyst for process efficiency. The hydrocarbons produced span the carbon range from C_7_ to C_24_, with a predominance of C_15_, C_16_, C_17_, and C_18_ compounds, which correspond to green diesel. These compounds were quantified by gas chromatography with flame ionization detection (GC-FID) using an external standard method, with results expressed as selectivity percentages.

In the HDT process of triacylglycerides, several simultaneous and consecutive reactions occur. Under hydrogen and high-temperature conditions, hydrogenolysis of triacylglycerides into fatty acids takes place, along with saturation of olefinic bonds and conversion of triacylglycerols to their corresponding fatty acids and propane. Subsequently, deoxygenation reactions involving fatty acids, methyl esters, fatty alcohols, and other oxygenated intermediates lead to the formation of hydrocarbons, while CO, CO_2_, H_2_O, propane, and light hydrocarbons are generated as byproducts through decarboxylation, decarbonylation, hydrodeoxygenation, hydrogenolysis, and secondary cracking pathways.^63,64^

To gain further insight into the deoxygenation pathways operating during vegetable oil hydrotreatment, the relative abundance of C_n_ and C_n‑1_ paraffins was evaluated from the GC-FID chromatograms of the most selective catalytic runs (RS4h and RF4h), as summarized in Table 11. In HDO, oxygen is removed as H_2_O while preserving the carbon chain length, leading primarily to the formation of C16 and C18 paraffins from C16 and C18 fatty acid derivatives present in the feedstocks. In contrast, decarboxylation and decarbonylation (DCO/DCO_2_) pathways remove one carbon atom as CO_2_ or CO, producing C15 and C17 paraffins.^53,65,66^ Since the fatty acid profiles of both feedstocks are dominated by C16 and C18 fatty acids (Table 5), the formation of C15 and C17 paraffins provides indirect evidence that oxygen removal occurred through decarboxylation and decarbonylation pathways, in which one carbon atom is released as CO_2_ or CO.

**Table 11:** Relative Contribution of C_n_ and C_n‑1_ Paraffins Obtained from GC-FID Analysis of the Most Selective Catalytic Runs

Sample	C15 area	C16 area	C17 area	C18 area	(C15+C17)/(C16+C18)
RS4h	109604	338586	721926	2406737	0.303
RF4h	110998.3	476295.9	579431.6	2463704	0.235

The calculated (C15+C17)/(C16+C18) ratios were 0.303 and 0.235 for SO (RS4h) and FO (RF4h), respectively. Since both values are significantly lower than unity, the results indicate that hydrodeoxygenation was the predominant deoxygenation pathway under the evaluated conditions.^53,65,66^ The slightly lower ratio observed for FO may indicate a somewhat greater contribution of HDO relative to SO. However, the difference between the two feedstocks was relatively small, suggesting that both oils exhibited a similar dominant deoxygenation behavior under the investigated conditions.

The feedstock characterization data presented in Table 4 showed that FO contained a higher concentration of free fatty acids (1.49%) than SO (0.88%), reflecting the degradation reactions that occur during the repeated frying cycles. Nevertheless, only a modest variation in the (C15+C17)/(C16+C18) ratio was observed between the two feedstocks (0.235 for FO and 0.303 for SO). This result suggests that the differences in feedstock composition exerted only a limited influence on the relative contributions of HDO and DCO/DCO_2_ pathways under the hydrogen-rich conditions employed in this study. In both cases, the predominance of HDO is inferred from the higher relative abundance of C16+C18 paraffins.

In addition, hydrocarbons lighter than the green diesel range (C5–C15) were detected in the product mixtures, indicating the occurrence of secondary hydrocracking reactions. As discussed later in the experimental design section, the formation of these lighter hydrocarbons became more pronounced at higher reaction temperatures, consistent with the enhanced cleavage of C–C bonds under more severe reaction conditions.^66^

The products from the control reactions and those with catalyst reduced for 2 and 4 h (RS0, RS2h, and RS4h, respectively), presented in Table 9, were further characterized by infrared spectroscopy (FTIR) and compared with the original SO, revealing chemical modifications resulting from the process, as illustrated in Figure 5.

**Figure 5:**
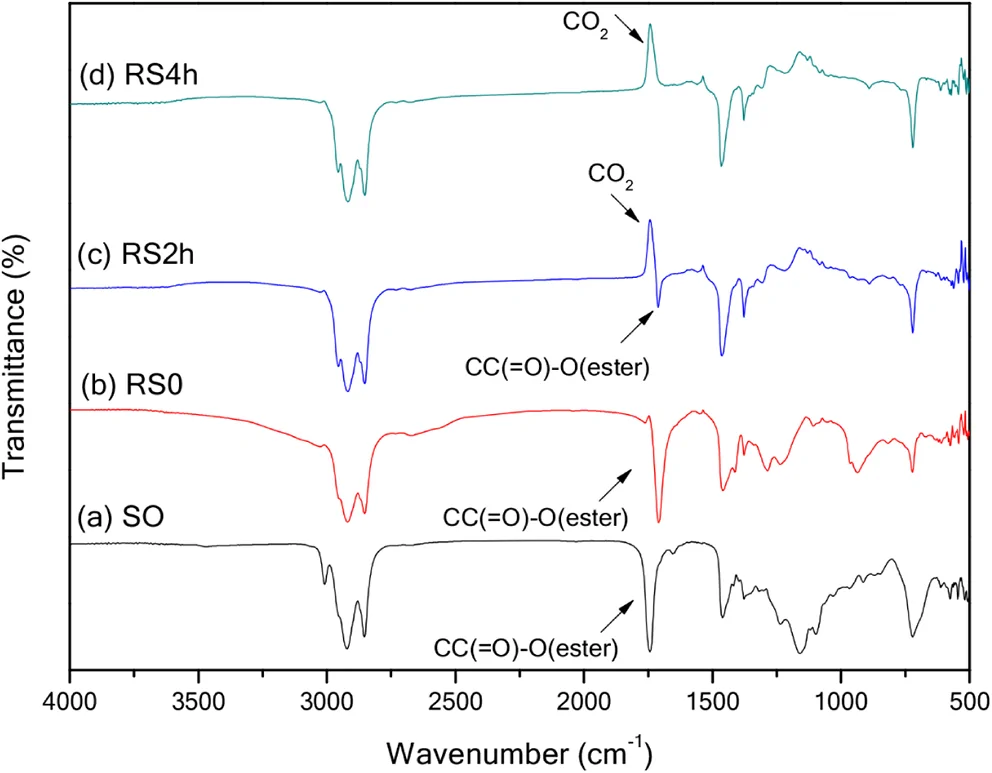
Infrared spectra of (a) soybean oil (SO) and the products of (b) control reaction (RS0), reactions performed with the catalyst (c) NiMoS_2_/Al_2_O_3_ reduced for 2 h (RS2h), and (d) NiMoS_2_/Al_2_O_3_ reduced for 4 h (RS4h).

The most notable difference between the infrared spectrum of SO (a) and that of the RS4h product (d) is the absence of the characteristic ester carbonyl stretching band at 1750 cm^–1^ in spectrum (d). In spectrum (a), bands at 1200 cm^–1^, corresponding to C–C­(=O)–O axial deformation in esters, and at approximately 1183 cm^–1^, likely associated with asymmetric stretching of the O–C–C bond, are observed. These bands are still present in spectra (b) and (c) but are no longer observed in spectrum (d), confirming the hydrodeoxygenation of the crude oil.

All spectra exhibit absorption in the 3000–2800 cm^–1^ region, corresponding to C–H stretching vibrations, typically attributed to methylene (CH_2_) and methyl (CH_3_) groups, both in hydrocarbon chains and in oxygenated compounds such as fatty acids.

A band at 1744 cm^–1^, possibly associated with carbonyl species or CO_2_ dissolved in the reaction medium, is observed in spectra (c) and (d), but not in the control reaction (b) nor in the original SO (a). This suggests the formation of CO_2_ through decarboxylation reactions. Although CO may also have been generated via decarbonylation, it was not detected in the FTIR spectra. At this stage, gaseous products were quantified only as mass percentages, as presented in Tables 8, 9, and 10. Their composition is likely a mixture of CO, CO_2_, C_3_H_8_, CH_4_, and H_2_O.

To complement the spectroscopic evidence and provide a global validation of the catalytic system, representative mass balances were established for the most selective catalytic runs (RS4h and RF4h), as summarized in Table 12. Near-complete mass closure was achieved (99.50–100.00%), confirming excellent consistency between the quantified liquid products and the overall material balance.

**Table 12:** Representative Mass Balances for the Catalytic Hydrodeoxygenation of Soybean Oil and Frying Oil under Optimum Conditions

Stream	RS4h	RF4h
Initial oil mass (g)	40.20	39.50
Total liquid products (g)	31.34	35.50
Gaseous products (g)^a^	8.66	4.00
Mass closure (%)	99.50	100.00

a Calculated by difference because of the absence of direct gas-phase collection in the batch reactor configuration.

Gaseous products were estimated by mass balance closure due to the absence of direct gas-phase collection in the batch reactor configuration. Gas formation is associated with decarboxylation and decarbonylation reactions (CO_2_ and CO formation), hydrogenolysis of the glycerol backbone (C_3_H_8_ formation), and secondary cracking reactions producing light hydrocarbons (C_1_–C_4_).^16,33,35^ In addition, water is expected to be generated mainly through the hydrodeoxygenation pathway, in which oxygen is removed from fatty acids as H_2_O while preserving the carbon chain length of the corresponding hydrocarbons.^10,14,15^ Although water was not quantified separately, since it condensed together with the liquid products, its formation is consistent with the predominance of deoxygenation pathways inferred from FTIR results and liquid-phase composition trends. The high mass closure suggests that major unaccounted losses were limited under the investigated conditions. Nevertheless, direct quantification of gaseous products, water formation, and coke deposition would be required for a rigorous assessment of material closure.

To better understand the catalytic activity of NiMoS_2_/Al_2_O_3_ in the HDO process of vegetable oils, the catalyst used in the SO reactions was characterized after use in the RS4h reaction, as presented in Table 13.

**Table 13:** Textural Characteristics of NiMoS_2_/Al_2_O_3_ Reduced for 4 h and NiMoS_2_/Al_2_O_3_ after Reaction with 40 g of Soybean Oil at 370 °C, 70 bar of H_2_, 800 rpm, 3% (m/m) Catalyst for 5 h^a^

Catalyst	S_BET_ (m^2^ g^–1^)	V_pores_ (cm^3^ g^–1^)	D_pores_ (nm)
NiMoS_2_/Al_2_O_3 reduced 4 h_	84	0.27	11.80
NiMoS_2_/Al_2_O_3 after first reaction_	87	0.19	6.03

a S_BET_: surface area (m^2^ g^–1^); V_pores_: average pore volume (cm^3^ g^–1^); D_pores_: average pore diameter (nm).

Figure 6(a) presents the typical X-ray diffraction (XRD) pattern of Al_2_O_3_ (cubic structure), with its main diffraction peaks observed at 2θ = 37°, 46°, and 67°, in agreement with the literature.^50^ The pattern shown in Figure 6(b) was previously discussed in Figure 4(b). In contrast, the diffractogram of the catalyst after the hydroprocessing reaction with SO, shown in Figure 6(c), reveals a decrease in the intensity of the peaks associated with the NiMoS and MoS_2_ phases, indicating a loss of crystallinity of the active phases. Additionally, the peaks at 2θ = 41° and 66° are attributed to carbonaceous phases and nickel aluminates, respectively. This suggests possible catalyst deactivation associated with the formation of nickel aluminate species and carbon deposition.^62–64^

**Figure 6:**
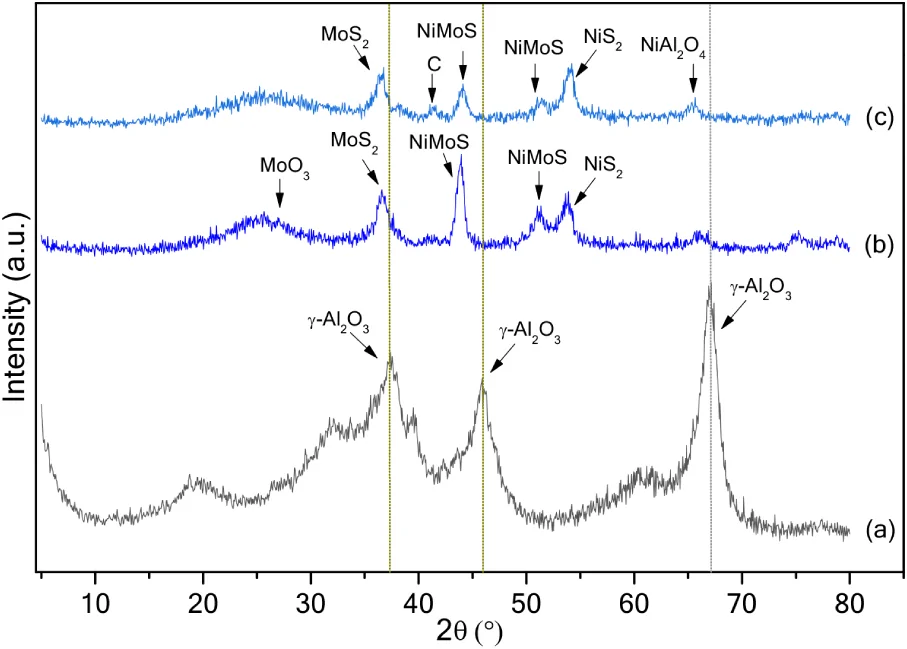
X-ray diffractograms of the catalyst (a) Al_2_O_3_, (b) NiMoS_2_/Al_2_O_3_ reduced for 4 h, and (c) NiMoS_2_/Al_2_O_3_ after reaction with 40 g of soybean oil at 370 °C, 70 bar of H_2_, 800 rpm, and 3% (w/w) catalyst for 5 h.

The comparison between the XRD pattern of the support (Al_2_O_3_) and those of the fresh and used catalysts reveals that the diffraction peaks of the catalyst overlap with the main peaks of Al_2_O_3_. This overlap arises from the high concentration of active phases (NiMoS) impregnated on the support, suggesting that the active phase responsible for the HDO process is located on the catalyst surface. However, low metal dispersion favors the agglomeration of crystalline phases on the surface, which, in turn, attenuates or eliminates the main diffraction peaks of the Al_2_O_3_ support. High levels of crystallinity may negatively impact the catalytic activity and stability of the material at high temperatures.^55^

Table 13 presents the results of the textural analysis of the NiMoS_2_/Al_2_O_3_ catalyst after 4 h of reduction and after its first use in SO hydroprocessing.

The NiMoS_2_/Al_2_O_3_ catalyst, reduced for 4 h and passivated prior to the reaction, exhibited a specific surface area of 84 m^2^ g^–1^. The textural analysis indicated an average pore diameter of 11.8 nm, classifying the material as predominantly mesoporous (2–50 nm), which is favorable for the diffusion and conversion of large molecules such as triacylglycerides. After the hydroprocessing reaction, the average pore diameter decreased to 6.03 nm, while still maintaining its mesoporous character. This reduction in pore volume and diameter suggests partial blockage, possibly due to the deposition of heavy intermediates or coke. Nevertheless, the catalyst may potentially be regenerated by recalcination followed by a new reduction step, enabling its reuse in HDO processes.

The NiMoS_2_/Al_2_O_3_ catalyst used in this study was supplied by the Centro de Pesquisa, Desenvolvimento e Inovação Leopoldo Américo Miguez de Mello (CENPES) and was selected due to its commercial relevance and industrial applicability. This material is widely used in petroleum refineries for the hydrotreatment of diesel and aviation fuels, aiming at the removal of sulfur-, nitrogen-, and oxygen-containing compounds. The sulfiding step plays a fundamental role in catalytic performance, as sulfided catalysts exhibit higher activity compared to their nonsulfided counterparts. The importance of this step in increasing conversion and selectivity in hydrotreated vegetable oil (HVO) processes is well established in the literature.^67^ According to Li (2018), sulfided catalysts are generally more effective than nonsulfided ones in promoting the conversion of vegetable oils into hydrocarbons due to their higher catalytic activity. However, despite their superior performance, these catalysts may cause undesirable sulfur contamination in the final products as a result of sulfur leaching during the reaction.^67,68^

To place the present results in the context of industrial operation and highlight the limitations associated with batch reactor studies, a brief comparison with typical continuous-flow hydrotreatment conditions is provided below. Although the batch experiments employed a catalyst/oil ratio of 3 wt % and a reaction time of 5 h, these conditions are more severe than those commonly encountered in industrial trickle-bed hydrotreatment units.^69,70^ Commercial renewable diesel processes are typically operated continuously at WHSV values in the range of approximately 0.5–2.0 h^–1^ and with lower catalyst inventories relative to the feed.^70,71^ Therefore, the present conditions should be regarded as laboratory-scale conditions for comparative evaluation of catalyst activity and selectivity, rather than direct industrial simulation.

### 3.4 Statistical Experimental Design

To investigate the influence of temperature, hydrogen pressure, and stirring speed on selectivity toward hydrocarbons (HCs) and green diesel (GD), a full factorial design (2^3^) with three central points was employed. The central points allowed for the evaluation of the curvature of the response surface and the estimation of the experimental error. This design enabled a comprehensive analysis of both main effects and interactions, providing insights into how these variables affect selectivity individually and in combination.

In this study, the HDO of SO was conducted using a commercial NiMoS_2_/Al_2_O_3_ catalyst under different conditions of temperature (350, 360, and 370 °C), hydrogen pressure (50, 60, and 70 bar), and stirring speed (500, 650, and 800 rpm). The selectivities toward hydrocarbons and green diesel obtained in the hydrotreatment of SO and FO are presented in Tables 14 and 15, respectively.

**Table 14:** Results Obtained from the 2^3^ Full Factorial Experimental Design with the Addition of Three Central Points for the Hydrotreatment of Soybean Oil (SO), Using the Commercial NiMoS_2_/Al_2_O_3_ Catalyst^a^

Reaction	T (°C)	P (bar)	Stirring (rpm)	X_TG_ (%)	S_HC_ (%)	S_GD_ (%)	S_C5–C15_ (%)	S_Others_ (%)
1	350	50	500	99.36	82.49	77.97	4.52	17.51
2	370	50	500	99.44	96.29	89.58	6.71	3.71
3	350	70	500	99.39	91.96	87.30	4.66	8.04
4	370	70	500	99.48	97.22	89.81	7.41	2.78
5	350	50	800	99.33	75.24	72.43	2.81	24.76
6	370	50	800	99.44	94.89	85.28	9.61	5.11
7	350	70	800	99.46	96.40	91.06	5.34	3.60
**8**	**370**	**70**	**800**	**99.50**	**97.91**	**93.74**	**4.17**	**2.09**
9	360	60	650	99.48	92.37	86.88	5.49	7.63
10	360	60	650	99.48	94.12	89.63	4.49	5.88
11	360	60	650	99.50	91.23	88.13	3.10	8.77

a X_TG_: triacylglyceride conversion; S_HC_: selectivity toward liquid hydrocarbons; S_GD (C16–C22)_: selectivity toward hydrocarbons in the green diesel (GD) range (C16–C22); S_Others_: selectivity toward other products (a mixture of free fatty acids, fatty alcohols, methyl esters, and waxes).

**Table 15:** Results Obtained from the 2^3^ Full Factorial Experimental Design with the Addition of Three Central Points for the Hydrotreatment of Frying Oil (FO), Using the Commercial NiMoS_2_/Al_2_O_3_ Catalyst^a^

Reaction	T (**°**C)	P (bar)	Stirring (rpm)	X_TG_ (%)	S_HC_ (%)	S_GD_ (%)	S_C5–C15_ (%)	S_Others_ (%)
1	350	50	500	98.84	82.26	79.35	2.91	17.74
2	370	50	500	98.92	96.06	90.96	5.10	3.94
3	350	70	500	98.87	91.73	88.68	3.05	8.27
4	370	70	500	98.96	97.15	91.19	5.96	2.85
5	350	50	800	98.81	75.01	73.81	1.20	24.99
6	370	50	800	98.92	94.66	86.66	8.00	5.34
7	350	70	800	98.94	96.17	92.44	3.73	3.83
**8**	**370**	**70**	**800**	**98.98**	**97.68**	**95.12**	**2.56**	**2.32**
9	360	60	650	98.96	92.14	86.58	5.56	7.86
10	360	60	650	98.96	93.89	88.28	5.61	6.11
11	360	60	650	98.98	91.00	89.51	1.49	9.11

a X_TG_: triacylglyceride conversion; S_HC_: selectivity toward liquid hydrocarbons; S_GD_: selectivity toward hydrocarbons in the green diesel (GD) range (C16–C22); S_C5–C15_: selectivity toward hydrocarbons in the biojet fuel range (C5–C15) and lighter hydrocarbons; S_Others_: selectivity toward other products (a mixture of free fatty acids, fatty alcohols, methyl esters, and waxes).

The analysis of Tables 14 and 15 shows that, in addition to the target hydrocarbons, the NiMoS_2_/Al_2_O_3_ catalyst also promoted the conversion of SO and FO into byproducts such as free fatty acids, fatty alcohols, fatty acid esters (methyl esters), and wax esters. Both feedstocks exhibited high selectivity toward total hydrocarbons, with values exceeding 97% under the most favorable conditions (Experiment 8), along with high selectivity toward hydrocarbons in the green diesel range. These results indicate that FO is also a viable feedstock for green diesel production via hydrotreatment. Besides the hydrocarbons within the green diesel range (C16–C22), smaller amounts of C5–C15 compounds were also detected. These lighter products are attributed mainly to secondary hydrocracking reactions occurring at elevated temperatures. The increase in temperature enhances C–C bond cleavage and favors the formation of shorter-chain hydrocarbons, explaining the increase in S_C5–C15_ observed in some experiments conducted at 370 °C.^24,38^

No significant formation of branched paraffins was observed in the chromatographic analyses. This behavior is consistent with the predominantly hydrogenation/deoxygenation character of sulfided NiMoS_2_/Al_2_O_3_ catalysts and with the limited bifunctional acidity of γ-Al_2_O_3_, which does not strongly promote skeletal isomerization. Consequently, the products obtained consisted predominantly of linear paraffins.^38,72^

It is observed that higher temperature and hydrogen pressure conditions result in higher selectivities toward hydrocarbons and green diesel, indicating the predominance of deoxygenation pathways under more severe reaction conditions.

The conversion of triacylglycerides (X_TG_, %) was also satisfactory for both oils, reaching 99.50% for the SO and 98.98% for the FO. To confirm which conditions of temperature, H_2_ pressure, and stirring speed led to higher selectivity toward hydrocarbons, a statistical analysis of the data was performed. Because triacylglyceride conversion (X_TG_, %) remained above 98% in all experiments and exhibited only a narrow variation range, it was not considered to be a suitable response for factorial modeling. Therefore, the statistical analysis focused on S_HC_ and S_GD_, which showed a greater sensitivity to the operating variables and represented the main performance indicators of the process.

#### 3.4.1 Statistical Analysis—SO

Based on the results of the experimental design for HDO reactions of SO (Table 14), the relationship between the response variables—hydrocarbon selectivity and C16–C22 hydrocarbon selectivity—and the studied variables—temperature, H_2_ pressure, and stirring speed—was established. Among the models available in Design-Expert 9.06, the linear model, determined by the least-squares method, proved to be the most suitable for fitting the experimental dataset.

Figure 7 and Table 16 present the estimated effects (Pareto chart) and regression coefficients (regression table) of the independent variables temperature (°C), stirring (rpm), and H_2_ pressure (bar) on hydrocarbon (HC) and S_GD_, considering the linear model, as well as the interactions between variables when NiMoS_2_/Al_2_O_3_ was used as the catalyst.

**Figure 7:**
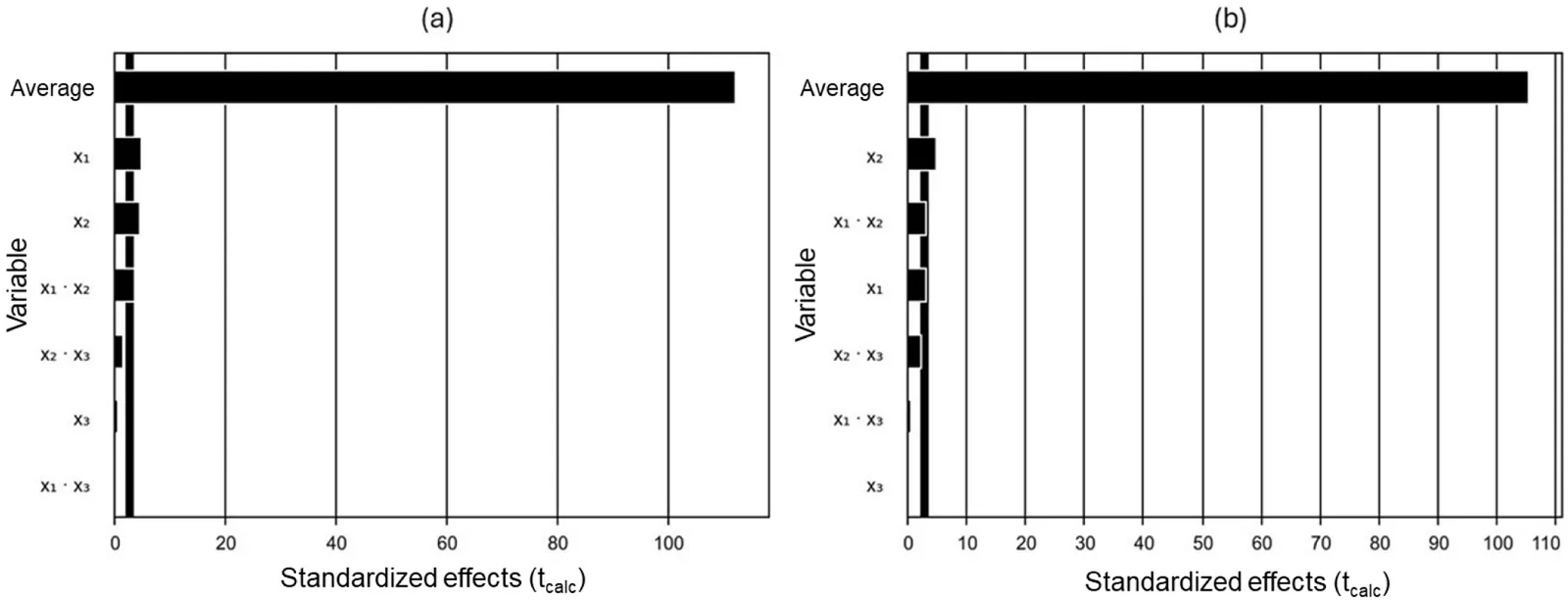
Pareto diagram of the standardized effects for (a) selectivity to S_HC_ hydrocarbons (%) and (b) selectivity to S_GD_ green diesel and lubricants (%) using NiMoS_2_/Al_2_O_3_ as the catalyst. X1: temperature (°C); X2: H_2_ pressure (bar); and X3: stirring.

**Table 16:** Regression Analysis of Coefficients for (a) Hydrocarbon Selectivity and (b) Green Diesel Range Selectivity When Soybean Oil (SO) Was Hydrotreated in the Presence of the Commercial Catalyst, NiMoS_2_/Al_2_O_3_

Name	Coefficients	Standard error	Calculated-*t*	*p*-value
**(a) HDT SO—Hydrocarbon selectivity**				
Mean	91.83	0.64	143.40	<0.05
Temperature, °C (X1)	5.03	0.75	6.69	<0.05
Pressure of H_2_, bar (X2)	4.32	0.75	5.76	<0.05
Stirring, rpm (X3)	–0.44	0.75	–0.58	0.59
X1·X2	–3.33	0.75	–4.44	0.06
X1·X3	0.26	0.75	0.35	0.74
X2·X3	1.72	0.75	2.29	0.08
**(b) HDT SO —Green diesel range selectivity**				
Mean	86.53	0.62	145.09	<0.05
Temperature, °C (X1)	3.71	0.70	5.30	<0.05
Pressure of H_2_, bar (X2)	4.58	0.70	6.55	<0.05
Stirring, rpm (X3)	–0.27	0.70	–0.38	0.72
X1·X2	–2.41	0.70	–3.44	0.09
X1·X3	0.18	0.70	0.25	0.81
X2·X3	2.19	0.70	3.13	<0.05

Based on the *p*-value criterion (≤0.05), an independent variable was considered significant in influencing hydrocarbon selectivity (%). In this study, temperature (X_1_) and hydrogen pressure (X_2_) were identified as significant factors, both exerting positive effects, with regression coefficients of 5.03 and 4.32, respectively. The sign of the coefficients indicates that positive values increase hydrocarbon selectivity, while negative values have a detrimental effect. Specifically, increasing temperature (350–370 °C) and hydrogen pressure (50–70 bar) favors the cleavage of oxygenated bonds and promotes hydrogenation by overcoming activation barriers. Additionally, higher hydrogen pressures stabilize reaction intermediates and increase hydrogen availability on the catalyst surface, thereby improving selectivity, although high temperatures may also lead to the formation of undesirable byproducts.^73–77^

On the other hand, stirring speed did not show a statistically significant influence, suggesting that under the evaluated experimental conditions, the reaction rate was governed by kinetics rather than mass transfer limitations, since increased agitation minimizes diffusion constraints.^74,76,77^ It is noteworthy that no significant interactions between the independent variables were observed regarding the hydrocarbon selectivity. Figure 9 presents a Pareto diagram confirming that temperature and hydrogen pressure are the only statistically significant factors influencing the system. The results indicate that the simultaneous increase in temperature and gas pressure favors hydrocarbon selectivity, which is in agreement with expected trends.

The validation of the models for HC and GD selectivity using the commercial NiMoS_2_/Al_2_O_3_ catalyst was performed through analysis of variance (ANOVA), as presented in Table 17, as well as through the *F*-distribution, with two *F*-tests conducted at this stage (Equation 3 and Equation 4).

**Table 17:** Results Obtained After Analysis of Variance (ANOVA) for the Response Variable: (a) Selectivity for Hydrocarbons, S_HC_ (%) and (b) Selectivity to Green Diesel, S_GD_ (%)^a^

Variation source	SS	FD	MS	F1_Cal_	*p*-value
**(a) HDT SO—hydrocarbon selectivity**					
Regression	446.50	6	77.75	17.2	<0.05
Residuals	18.04	4	4.5	**F2**_**Cal**_	
Lack of fit	13.80	2	6.90	3.3	0.23
Pure error	4.24	2	2.12		
Total	484.53	10			
R^2^ = 0.9628					
$$R_{\mathrm{adj}}^{2}=0.9070$$					
**(b) Selectivity of HDT SO—green diesel**					
Regression	363.45	6	60.57	15.5	<0.05
Residuals	15.65	4	3.91	**F2**_**Cal**_	
Lack of fit	11.86	2	5.93	3.1	0.24
Pure error	3.80	2	1.89		
Total	379.10	10			
R^2^ = 0.9235					
$$R_{\mathrm{adj}}^{2}=0.8088$$					

a SS: sum of squares; FD: freedom degrees; MS: mean squares.

The values of F1 and F2 were calculated using Equation 3 and Equation 4.

**• F1 Test:**

To evaluate the significance of the linear regression, the null hypothesis (H_0_)—which assumes that all regression coefficients are equal to zero and that the slope of the fitted line is null—was tested using the F1 test. For H_0_ to be rejected, the calculated F value (F1_Cal_, Equation 3) must exceed the critical tabulated value (F1_tab_), and the *p*-value must be statistically significant (≤0.05). As shown in Table 15, the *p*-values for both models are below the predefined significance level (α = 0.05), indicating statistical relevance. Moreover, based on the critical F values reported by Rodrigues and Iemma (2014), the F1_Cal_ value for the S_HC_ (%) model is 17.2, approximately 2.8 times higher than F1_tab_ (F1_tab_ = F(6.4;0.05) = 6.16).^78^ A similar result is observed for the S_GD_ (%) model, where F1_Cal_ is 15.5, approximately 2.5 times higher than the critical value. Therefore, the null hypothesis can be rejected in both cases, confirming the existence of a significant linear relationship between the response variables (S_HC_ (%) and S_GD_ (%)) and the independent variables.

3 $$F1_{\mathrm{Cal}(6,4)}=\frac{MS_{\mathrm{Regression}}}{MS_{\mathrm{Residual}}}$$

**• F2 Test:**

To evaluate the goodness of fit of the models, the lack-of-fit F2 test was applied, in which model adequacy is confirmed when the calculated F2 value (F2_Cal_, Equation 4) is lower than the tabulated value (F2_tab_), considering a 95% confidence level, and when the *p*-value is not statistically significant (>0.05). According to Table 15, the *p*-values for the S_HC_ (%) and S_GD_ (%) models are 0.23 and 0.24, respectively, both above the 0.05 threshold. Additionally, the calculated F2 values for the S_HC_ and S_GD_ models (3.3 and 3.1, respectively) are lower than the critical value (F2_tab_ = F(2;2;0.05) = 19.00) reported by Rodrigues and Iemma (2014).^78^ These results indicate that the null hypothesis—according to which the models adequately represent the data—cannot be rejected, thus confirming a satisfactory model fit.

4 $$F2_{\mathrm{Cal}(2,2)}=\frac{MS_{\mathrm{Lack of fit}}}{MS_{\mathrm{Pure error}}}$$

Additionally, high coefficients of determination were obtained for SO hydrotreatment with R^2^ values of 0.9628 and 0.9235 for S_HC_ and S_GD_, respectively. The corresponding adjusted coefficients ( $R_{\mathrm{adj}}^{2}$ = 0.9070 and 0.8088) also remained high, indicating that the fitted models retained good explanatory power after accounting for the number of model terms. Combined with the nonsignificant lack-of-fit results (*p* > 0.05), these findings confirm the adequacy of the proposed models and their satisfactory predictive capability.

The equations describing the responses for hydrocarbon selectivity (*Y*_1_, S_HC_, %) and green diesel selectivity (*Y*_2_, S_GD_, %) under SO hydrotreatment in the presence of the commercial NiMoS_2_/Al_2_O_3_ catalyst are presented in Equation 5 and Equation 6, respectively.

5 $$Y_{1}=91.83+5.03x^{1}+4.32x^{2}-0.44x^{3}-3.34x^{1}x^{2}+0.26x^{1}x^{3}+1.72x^{2}x^{3}$$

6 $$Y_{2}=86.53+3.71x^{1}+4.58x^{2}-0.27x^{3}-2.41x^{1}x^{2}+0.18x^{1}x^{3}+2.19x^{2}x^{3}$$

where:

*Y*_1_ = S_HC_ (%), using the NiMoS_2_/Al_2_O_3_ catalyst.

*Y*_2_ = S_GD_ (%), using the NiMoS_2_/Al_2_O_3_ catalyst.

*x* ^1^ = temperature (°C);

*x* ^2^ = H_2_ pressure (bar);

*x* ^3^ = stirring (rpm).

Figure 8 presents the comparison between experimental and predicted results for (a) HC selectivity (%) and (b) GD selectivity (%), using NiMoS_2_/Al_2_O_3_ as the catalyst.

**Figure 8:**
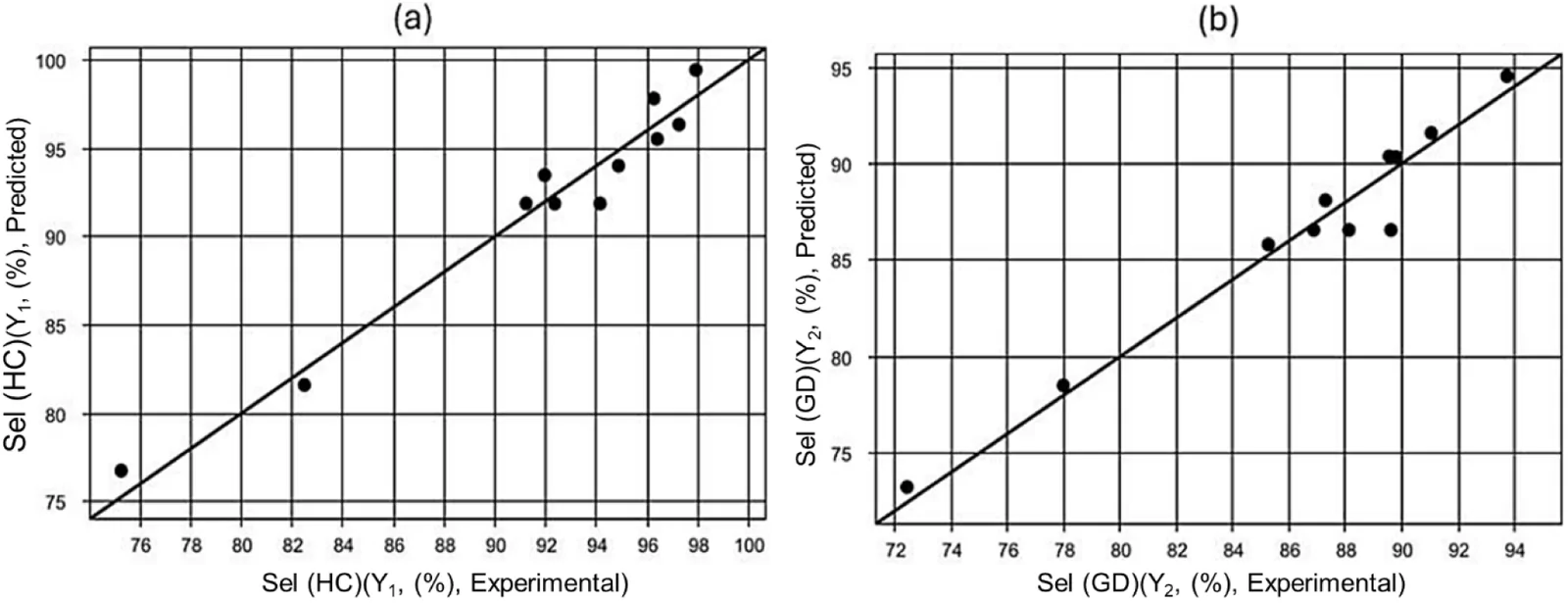
Graph of experimental results versus the results obtained by the proposed model for (a) hydrocarbon selectivity (S_HC_, %) and (b) green diesel selectivity (S_GD_, %), when soybean oil (SO) was hydrotreated in the presence of the commercial catalyst NiMoS_2_/Al_2_O_3_. The dots represent the experimental results (●), and the results obtained from the linear statistical model are represented by the line (−). The closer the dots are to the line, the closer the experimental results are to the model’s results.

In general, the experimental values of hydrocarbon and green diesel selectivity are closely distributed around the predicted lines, indicating good agreement with the model estimates.

Subsequently, the effects of reaction parameters on the response variables were evaluated using two-dimensional response surface plots (Figure 9), constructed from the model equations (Equation 5 and Equation 6). In these plots, green regions indicate the highest selectivities toward hydrocarbons and green diesel.

**Figure 9:**
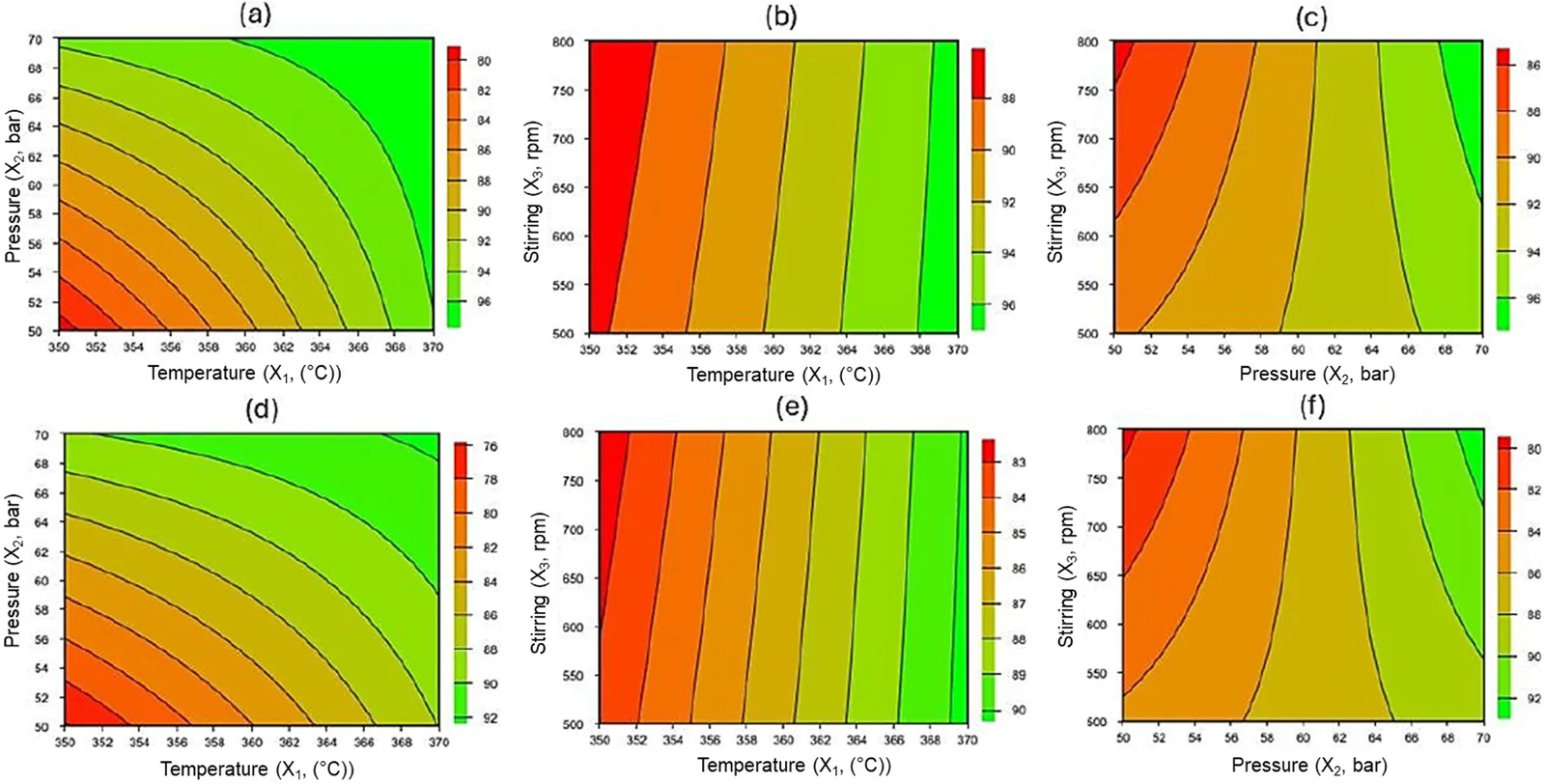
2D plots illustrating the selectivity to hydrocarbons (S_HC_, %) and green diesel (S_GD_, %) during the hydrotreatment of soybean oil (SO) using the commercial NiMoS_2_/Al_2_O_3_ catalyst. (a) temperature vs H_2_ pressure for S_HC_; (b) temperature vs stirring speed for S_HC_; (c) H_2_ pressure vs stirring speed for S_HC_; (d) temperature vs H_2_ pressure for S_GD_; (e) temperature vs stirring speed for S_GD_; (f) H_2_ pressure vs stirring speed for S_GD_.

Figure 9 shows the effects of temperature, stirring speed, and H_2_ pressure on HC and GD selectivity. Consistent with the statistical analysis, temperature and H_2_ pressure were the dominant factors, whereas stirring speed exhibited only a minor individual effect within the investigated range. In Figure 9(b) and 9(e), higher temperatures (360–370 °C) increase selectivity regardless of stirring speed, indicating a more pronounced influence of stirring at elevated temperatures. Figures 9(c) and 9(f) show that at low H_2_ pressures (<54 bar), increasing stirring speed reduces selectivity; however, this effect is reversed at higher pressures, where both HC (>96%) and GD (>92%) selectivities are favored by higher stirring speeds (>700 rpm) and pressures (>68 bar). Figures 9(a) and 9(d) further confirm that high temperatures (∼370 °C), combined with high H_2_ pressures (>65 bar), enhance HDO performance, resulting in selectivities above 90%. Thus, the optimal conditions for maximizing hydrocarbon selectivity in the green diesel range were identified as 370 °C, 70 bar H_2_, and 800 rpm, as observed in Experiment 8 (Table 14).

#### 3.4.2 Statistical Analysis—FO

Table 15 presents the experimental conditions and selectivities obtained for hydrocarbons and green diesel (GD), which were used as input data for the statistical analysis of FO hydrotreatment using the commercial NiMoS_2_/Al_2_O_3_ catalyst.

Figure 10 and Table 18 present the estimated effects (Pareto chart) and regression coefficients (regression table) of the independent variables (temperature (°C), H_2_ pressure (bar), and stirring speed (rpm)) on hydrocarbon selectivity (S_HC_, %) and green diesel selectivity (S_GD_, %), based on a linear model that also considers interactions between variables. These analyses were conducted for the hydrotreatment of FO using the commercial NiMoS_2_/Al_2_O_3_ catalyst.

**Figure 10:**
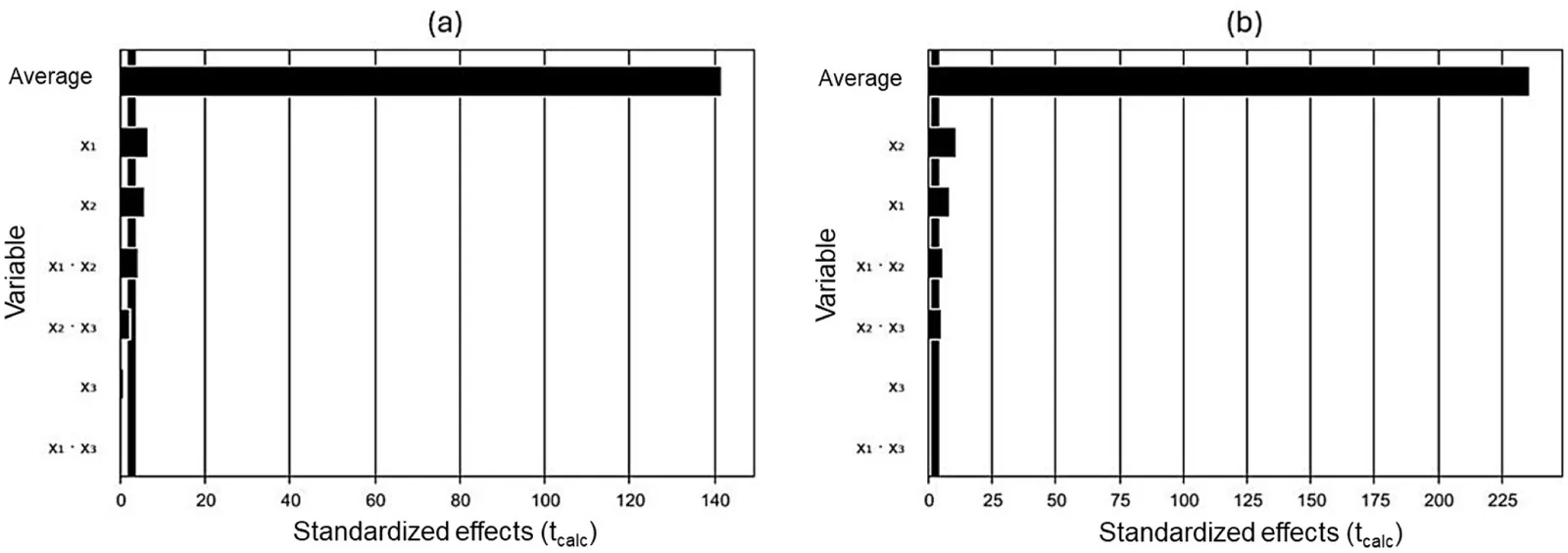
Pareto diagram of the magnitudes of the standard effects for (a) hydrocarbon selectivity and (b) S_GD_ when frying oil (FO) was hydrotreated in the presence of the commercial catalyst NiMoS_2_/Al_2_O_3_.

**Table 18:** Regression Analysis of Coefficients for (a) Hydrocarbon Selectivity and (b) S_GD_ When Frying Oil (FO) Was Hydrotreated in the Presence of the Commercial Catalyst, NiMoS_2_/Al_2_O_3_

Name	Coefficients	Standard error	Calculated-*t*	*p*-value
**(a) HDT FO—Hydrocarbon selectivity**				
Mean	91.61	0.65	141.897	<0.05
Temperature, °C (X1)	5.05	0.76	6.67	<0.05
Pressure of H_2_, bar (X2)	4.34	0.76	5.732	<0.05
Stirring, rpm (X3)	–0.46	0.76	–0.61	0.58
X1·X2	–3.31	0.76	–4.38	0.08
X1·X3	0.24	0.76	0.32	0.76
X2·X3	1.70	0.76	2.25	0.09
**(b) HDT FO—Green diesel selectivity**				
Mean	87.51	0.37	236.22	<0.05
Temperature, °C (X1)	3.71	0.43	8.53	<0.05
Pressure of H_2_, bar (X2)	4.58	0.43	10.55	<0.05
Stirring, rpm (X3)	–0.27	0.43	–0.62	0.57
X1·X2	–2.41	0.43	–5.54	0.07
X1·X3	0.18	0.43	0.40	0.70
X2·X3	2.19	0.43	5.04	<0.05

The statistical analysis of FO hydrotreatment, based on *p*-values (significance threshold ≤ 0.05), revealed that temperature and H_2_ pressure significantly influenced hydrocarbon selectivity (S_HC_, %), while stirring had no significant effect. As shown in Table 18(a), both temperature and H_2_ pressure exhibited positive and statistically significant effects, with regression coefficients of 5.05 and 4.34, respectively. These values indicate that increasing temperature from 350 to 370 °C and H_2_ pressure from 50 to 70 bar increased hydrocarbon selectivity by 5.05 and 4.34 units, respectively. In contrast, stirring speed, when evaluated independently, showed no significant impact on S_HC_ (%). Furthermore, interaction terms between independent variables were not statistically significant for hydrocarbon selectivity. These findings are corroborated by the Pareto chart (Figure 10a), confirming temperature and pressure as the dominant factors. Reaction 8 (Table 15) reinforces this trend, showing that higher temperatures and H_2_ pressures are associated with higher S_HC_ (%).

Regarding S_GD_, a similar pattern was observed. As shown in Table 18(b) and Figure 12(b), temperature and H_2_ pressure were again significant and positive factors, with regression coefficients of 3.71 and 4.58, respectively. This indicates that increasing temperature from 350 to 370 °C and H_2_ pressure from 50 to 70 bar led to increases in GD selectivity of 3.71 and 4.58 units, respectively. Although stirring speed alone was not significant for GD selectivity, its interaction with H_2_ pressure showed a positive and statistically significant effect, with a regression coefficient of 2.19. This suggests that higher stirring levels, combined with high pressures, favor green diesel production, as evidenced by reaction 8 (Table 15).

Overall, the behavior observed for FO consistently reproduces that found for SO, as expected, considering that the used cooking oil in Brazil is predominantly derived from soybean oil. These findings highlight the central roles of temperature and hydrogen pressure in maximizing product selectivity during hydrodeoxygenation.

The statistical validation of the predictive models for S_HC_ and S_GD_ during FO hydrotreatment over NiMoS_2_/Al_2_O_3_ was performed using ANOVA, as summarized in Table 19. This procedure included two *F*-tests based on the *F*-distribution (Equation 7 and Equation 8) to evaluate model significance and robustness.

**Table 19:** Statistical Analysis of Variance (ANOVA) for the Response Variables: (a) Selectivity for Hydrocarbons, S_HC_ (%) and (b) Selectivity for Green Diesel, S_GD_ (%)^a^

Variation source	SS	FD	MS	F1_Cal_	*p*-value
**(a) HDT FO—hydrocarbon selectivity**					
Regression	467.94	6	77.99	17.0	<0.05
Residuals	18.34	4	4.58	**F2**_**Cal**_	
Lack of fit	14.10	2	7.05	3.3	0.23
Pure error	4.24	2	2.12		
Total	486.28	10			
R^2^ = 0.9623					
$$R_{\mathrm{adj}}^{2}=0.9058$$					
**(b) HDT FO—green diesel selectivity**					
Regression	363.45	6	60.57	40.0	<0.05
Residuals	15.65	4	3.91	**F2**_**Cal**_	
Lack of fit	11.86	2	5.93	0.4	0.72
Pure error	3.80	2	1.89		
Total	379.10	10			
R^2^ = 0.9837					
$$R_{\mathrm{adj}}^{2}=0.9592$$					

a SS: sum of squares; FD: freedom degrees; MS: mean squares.

The comparison between calculated and critical F-values indicates that the statistical criteria for F1 and F2 are satisfied, confirming model adequacy under the evaluated conditions. Furthermore, high coefficients of determination were obtained for FO hydrotreatment with R^2^ values of 0.9623 for S_HC_ and 0.9837 for S_GD_. The corresponding adjusted coefficients ( $R_{\mathrm{adj}}^{2}$ = 0.9058 and 0.9592, respectively) also remained high, indicating that the models retained excellent explanatory power after correction for the number of fitted terms. Combined with the nonsignificant lack-of-fit results (*p* > 0.05), these findings confirm the adequacy and predictive capability of the proposed models.

The mathematical models for S_HC_ (*Y*_1_) and S_GD_ (*Y*_2_) are presented in Equation 7 and Equation 8, respectively.

7 $$Y_{1}=91.61+5.05x^{1}+4.34x^{2}-0.46x^{3}-3.31x^{1}x^{2}+0.24x^{1}x^{3}+1.70x^{2}x^{3}$$

8 $$Y_{2}=87.51+3.71x^{1}+4.58x^{2}-0.27x^{3}-2.41x^{1}x^{2}+0.18x^{1}x^{3}+2.19x^{2}x^{3}$$

where:

*Y*_1_ = S_HC_ (%), using the NiMoS_2_/Al_2_O_3_ catalyst;

*Y*_2_ = S_GD_ (%), using the NiMoS_2_/Al_2_O_3_ catalyst;

*x* ^1^ = temperature (°C);

*x* ^2^ = H_2_ pressure (bar);

*x* ^3^ = stirring (rpm).

Figure 11 presents the correlation plots between experimental data and model predictions for S_HC_(%) and S_GD_(%) during FO hydrotreatment using the commercial NiMoS_2_/Al_2_O_3_ catalyst.

**Figure 11:**
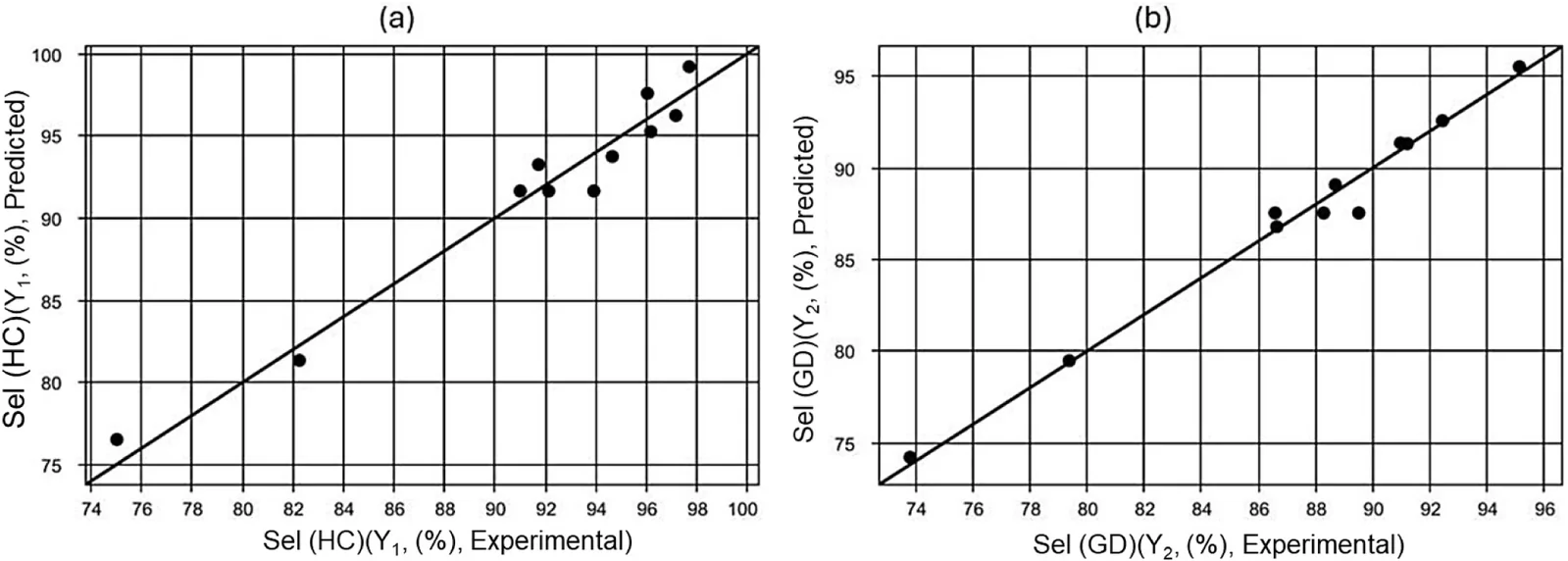
Graphs comparing experimental data and values predicted by the proposed model for (a) hydrocarbon selectivity (S_HC_, %) and (b) green diesel selectivity (S_GD_, %) during the hydrotreatment of frying oil (FO) over the commercial NiMoS_2_/Al_2_O_3_ catalyst. Experimental data points are represented by dots (●), while the predictions from the linear statistical model are indicated by the solid line (−). The proximity of the data points to the line reflects the degree of agreement between the experimental results and the model predictions.

The comparison between experimental data and model predictions shows that the experimental points closely follow the proposed linear trends, indicating a good fit and a strong agreement between experimental results and predicted values.

Based on the model equations (Equation 7 and Equation 8), two-dimensional response surface plots were constructed to illustrate the effects of the independent variables—temperature (350–370 °C), hydrogen pressure (50–70 bar), and stirring speed (500–800 rpm)—on the dependent variables: hydrocarbon selectivity (S_HC_, %) and green diesel selectivity (S_GD_, %). These results, obtained from FO hydrotreatment over the commercial NiMoS_2_/Al_2_O_3_ catalyst, are presented in Figure 12.

**Figure 12:**
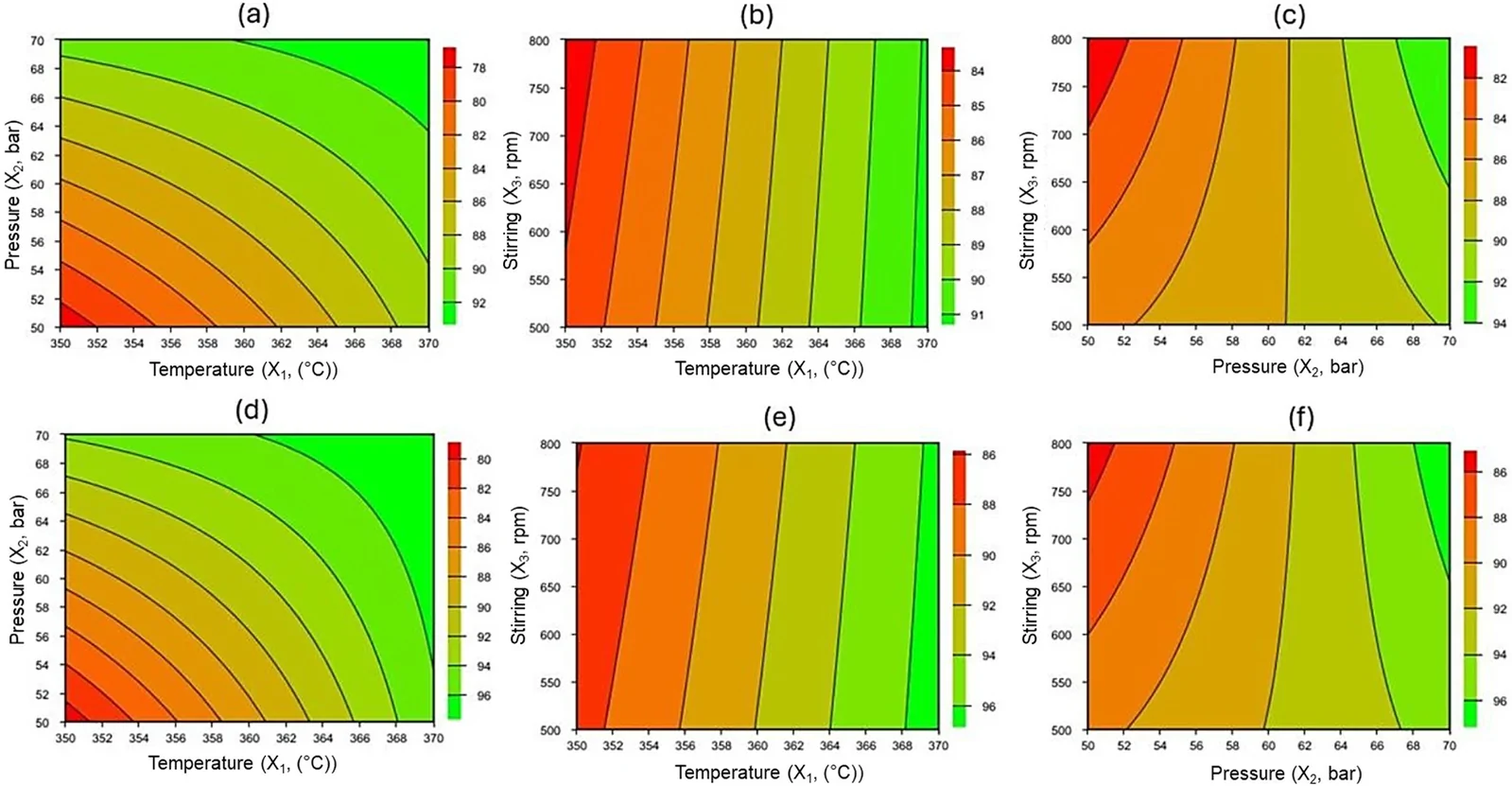
2D graphical representations of hydrocarbon selectivity (S_HC_, %) and green diesel selectivity (S_GD_, %) during the hydrotreatment of frying oil (FO) in the presence of the commercial NiMoS_2_/Al_2_O_3_ catalyst. (a) Temperature vs H_2_ pressure for S_HC_; (b) temperature vs stirring speed for S_HC_; (c) H_2_ pressure vs stirring speed for S_HC_; (d) temperature vs H_2_ pressure for S_GD_; (e) temperature vs stirring speed for S_GD_; (f) H_2_ pressure vs stirring speed for S_GD_.

The regions highlighted in green in Figure 12 indicate the experimental conditions under which the highest selectivity values were achieved, particularly the combination of high temperature (≈ 370 °C), high hydrogen pressure (70 bar), and high stirring rate (800 rpm). Consistently, the contour plots (Figure 12) demonstrate that temperature and H_2_ pressure are the most influential factors for both responses, as confirmed by the analysis of the coefficients of the fitted model, which exhibit the highest absolute values for these variables. Increasing temperature raises the kinetic energy of the system, promoting more frequent and more effective molecular collisions and, consequently, favoring hydrocarbon formation. In contrast, reducing H_2_ pressure hinders mass transfer and limits hydrogen availability at the catalyst active sites, negatively impacting deoxygenation rates. Although the stirring rate proved to be statistically less significant as an isolated factor, its interaction with H_2_ pressure positively affected green diesel selectivity, especially at higher temperatures. Figures 12(b), (e), (c), and (f) show that under low H_2_ pressures (<54 bar), increasing stirring tends to reduce the formation of desired products; however, this trend is reversed at higher pressures (>68 bar), where selectivity values above 96% for S_HC_ and 92% for S_GD_ were observed. Overall, the graphical analyses suggest that the optimal conditions to maximize hydrocarbon selectivity within the range of interest correspond to temperatures between 350–370 °C, an H_2_ pressure of 70 bar, and a stirring rate of 800 rpm, with experiment 8 (370 °C, 70 bar, 800 rpm) standing out as the most effective in terms of both selectivity and liquid product yield (Table 20).

It should be emphasized that the highest selectivities were obtained at the upper boundary of the investigated experimental domain (370 °C, 70 bar H_2_, and 800 rpm). Therefore, the true optimum may lie outside the evaluated range, and further studies employing response-surface methodologies with expanded operating windows are recommended.

#### 3.4.3 Comparison of Catalytic Performance with Literature Reports

To further assess the performance of the commercial NiMoS_2_/Al_2_O_3_ catalyst under the optimum conditions in this study (370 °C, 70 bar H_2_, and 800 rpm), a quantitative comparison was conducted with representative literature reports on the hydrodeoxygenation of vegetable oils and waste cooking oils using both sulfided NiMo-based catalysts and noble-metal catalysts. The comparison includes feedstock type, reaction conditions, conversion or yield, and selectivity toward diesel-range hydrocarbons (C16–C22), allowing the catalytic performance achieved in the present work to be positioned within the current state of the art.

**Table 20:** Comparison of Catalytic Performance in Hydrodeoxygenation of Waste Oils

Catalyst	Feedstock	T (°C)	H_2_ (bar)	Performance metric (%)^a^	S_GD_ (%)^b^	Ref.
Commercial NiMoS_2_/Al_2_O_3_	FO	370	70	TAG conversion = 98.98	95.12	This work
0.2-NiMoS_2_/Al_2_O_3_	WSBO^c^	300	40	C15–C18 yield = 56.9	98.1	Prangklang et al. (2023)
Sulfided NiMo/Al_2_O_3_	WCO	350	70	Oil conversion = 99.8	90.7	Toba et al. (2011)
2.4%Ru/NbOPO_4_	WCO	350	30	Liquid yield = 78	96	Rosa et al. (2026)
Ir-ReOx/SiO_2_	WCO	180	20	Diesel-ranged alkane yield = 82 wt %	98.7^d^	Liu et al. (2018)

a Performance metrics vary among studies according to the methodology adopted by each author (e.g., feedstock conversion, hydrocarbon yield, or C15–C18 selectivity). Therefore, the table provides a comparative overview rather than a strict ranking of catalytic activity.

b S_GD_ denotes green diesel selectivity, defined as the selectivity toward liquid hydrocarbons in the green diesel range. Although most studies consider C16–C22 paraffins, some literature reports extend this range to C10–C20 hydrocarbons depending on the catalyst system and product classification criteria.

c WSBO: waste soybean oil.

d Estimated from reported product distributions, as green diesel selectivity was not explicitly provided in the original study.

Direct quantitative comparison among studies should be interpreted with caution because different authors report catalytic performance using distinct metrics, including feedstock conversion, hydrocarbon yield, liquid yield, and diesel range selectivity.

The commercial NiMoS_2_/Al_2_O_3_ catalyst employed in this work achieved 98.98% TAG conversion and 95.12% S_GD_ at 370 °C and 70 bar H_2_, representing a highly competitive performance among NiMo-based systems reported for waste-oil hydrodeoxygenation.

Literature NiMo catalysts exhibit similarly high selectivity, although often accompanied by lower overall hydrocarbon yields. For instance, Prangklang et al. reported 98.1% S_GD_ over 0.2-NiMoS_2_/Al_2_O_3_, corresponding to a C15–C18 hydrocarbon yield of 56.9%, indicating a trade-off between selectivity and total hydrocarbon production.^79^ In contrast, sulfided NiMo/Al_2_O_3_ reported by Toba et al. achieved nearly complete conversion (99.8%) but lower S_GD_ (90.7%) compared to the present study.^80^

Noble-metal catalysts also show high activity but under more complex or less directly comparable conditions. Ru/NbOPO_4_ reaches 78 wt % liquid yield with 96% S_GD_; however, the reaction proceeds predominantly via a decarboxylation/decarbonylation (DCOx) pathway, which involves intrinsic carbon loss and affects the effective carbon efficiency of the process.^81^ In addition, Ir–ReOx/SiO_2_ achieves 82 wt % diesel-range alkane yields only after extended reaction times (18 h) and in the presence of impurity-containing feedstocks, indicating slower overall kinetics and sensitivity to catalyst deactivation.^82^

Overall, despite differences in feedstock quality, reaction conditions, reaction pathways, and performance metrics, the results demonstrate that the NiMoS_2_/Al_2_O_3_ catalyst employed in this work provides an optimal balance between the TAG conversion and S_GD_. This performance places it within the upper range of reported catalytic systems for waste oil hydrodeoxygenation under industrially relevant conditions.

### 3.5 Reuse

Reuse experiments were carried out using the commercial catalyst NiMoS_2_/Al_2_O_3_ and vegetable oils (FO and SO) under the optimized conditions established in the experimental design (370 °C, 70 bar, 800 rpm, 5 h). At the end of each reaction cycle, the catalyst was separated from the reaction mixture by filtration and subjected to sequential washing with chloroform, isopropanol, and acetone. Subsequently, the catalyst was reused for four additional cycles under identical conditions. The results of these reuse experiments with the NiMoS_2_/Al_2_O_3_ catalyst in the hydrotreatment of FO and SO are summarized in Table 21.

**Table 21:** Results of Reuse Experiments, Reusing NiMoS_2_/Al_2_O_3_ as a Catalyst; Conditions: 800 rpm; 3% Catalyst Relative to the Mass of (a) Frying Oil and (b) Soybean Oil; 370 °C; 5 h, 70 bar^a^

**(a) FO**					**(b) SO**			
Cycle	X_TG_ (%)	S_HC_ (%)	S_GD_ (%)	S_C5–C15_ (%)	X_TG_ (%)	S_HC_ (%)	S_GD_ (%)	S_C5–C15_ (%)
1	99.03	97.22	92.73	7.27	99.23	97.22	92.73	7.27
2	94.45	96.06	93.20	6.79	95.48	97.09	93.30	6.70
3	93.26	94.77	92.48	7.51	94.37	96.18	92.61	7.39
4	93.42	95.01	86.91	13.08	93.96	96.15	87.05	12.95
5	93.83	94.39	84.32	15.67	94.33	95.26	84.45	15.55

a X_TG_ (%): triacylglyceride conversion; S_HC_ (%): hydrocarbon selectivity; S_GD_ (%): green diesel selectivity; S_C5–C15_ (%): aviation biokerosene selectivity.

Figure 13 presents plots correlating the trends in triacylglycerol conversion and selectivities obtained with FO and SO (Table 21) after each reuse experiment of the NiMoS_2_/Al_2_O_3_ catalyst.

**Figure 13:**
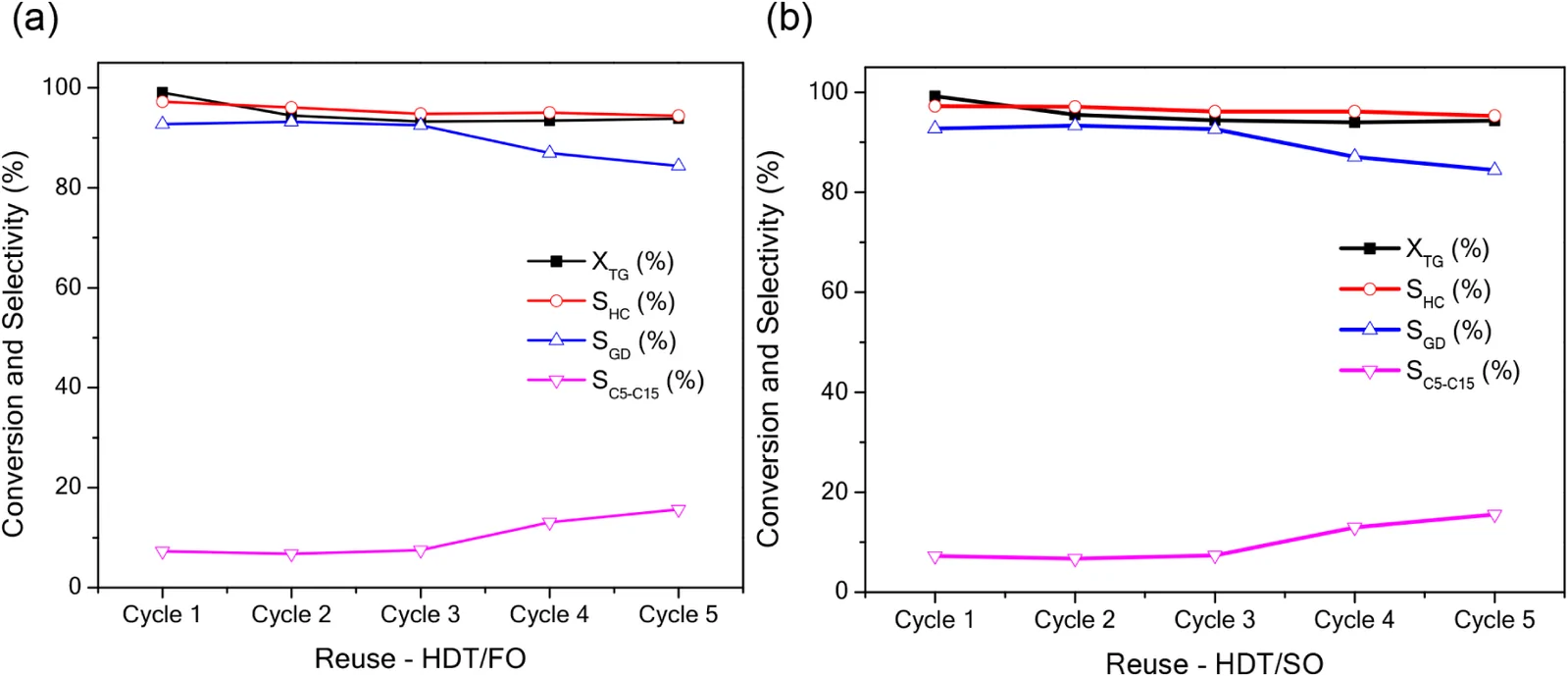
Triacylglyceride conversion behavior and selectivity to hydrocarbons (S_HC_, %), green diesel (S_GD_, %), and aviation biokerosene (S_C5–C15_, %) with (a) frying oil (FO) and (b) soybean oil (SO), reusing the NiMoS_2_/Al_2_O_3_ catalyst.

The NiMoS_2_/Al_2_O_3_ catalyst exhibited high initial catalytic activity, achieving triglyceride conversions (X_TG_) of 99.03% for FO (Table 21(a) and Figure 13(a)) and 99.23% for SO (Table 21(b) and Figure 13(b)). Between the first and second cycles, a moderate decrease in conversion to approximately 95% was observed, followed by stabilization up to the fifth cycle with average values of 93.51% (FO) and 94.22% (SO). This initial deactivation pattern is likely associated with the restructuring of the active sulfided phase, partial desulfurization, accumulation of carbonaceous deposits, and/or other deactivation mechanisms. However, the available experimental evidence does not allow the individual contributions of each mechanism to be quantitatively distinguished.^83–85^

The selectivity toward total hydrocarbons (S_HC_) remained high and consistent throughout the cycles, with average values of 95.49% (FO) and 96.38% (SO), suggesting the predominance of deoxygenation pathways and the partial preservation of the active Ni–Mo–S structure.^86^ However, from the third cycle onward, a decrease in S_GD_ was observed, concomitant with an increase in the C5–C15 fraction (S_C5–C15_). This shift toward hydrocracking reactions may be associated with gradual changes in the physicochemical properties of the bifunctional system, a phenomenon also reported by Wei et al. (2025) and Carrasco Díaz et al. (2024).^85,87^

From a mechanistic standpoint, the changes in product distribution and the loss of activity are likely associated with progressive modifications of the sulfided phase and partial pore blockage caused by the accumulation of carbonaceous species.^88^ Although these structural modifications affect the accessibility of active sites and the efficiency of hydrogenation and deoxygenation steps, the NiMoS_2_/Al_2_O_3_ catalyst demonstrated robustness, exhibiting similar performance for both oils used. Thus, the system proves to be a promising catalyst, maintaining high performance and hydrocarbon selectivity, even after multiple reuse cycles.

It should be emphasized that the catalyst was intentionally operated without sulfur cofeeding to simulate the processing of sulfur-free renewable feedstocks in conventional refinery infrastructure. Under these conditions, gradual desulfidation is expected and may contribute to the catalyst deactivation. Therefore, the observed performance reflects the catalyst robustness under realistic retrofit conditions rather than under continuously sulfided operations.

#### 3.5.1 Catalyst Characterization after Reuse Cycles

To investigate the catalyst deactivation observed at the end of the reuse experiments in HDT reactions with FO and SO, the spent catalyst was subjected to comprehensive textural characterization, as presented in Table 22.

**Table 22:** Textural Characteristics of the Commercial NiMoS_2_/Al_2_O_3_ after Five Cycles of Reuse in Hydrotreating (HDT) Reactions with Frying Oil (FO) and Soybean Oil (SO)^a^

Catalyst	S_BET_ (m^2^ g^–1^)	V_pores_ (cm^3^ g^–1^)	D_pores_ (nm)
NiMoS_2_/Al_2_O_3 reduced_	84	0.27	11.8
NiMoS_2_/Al_2_O_3 reuse FO_	34	0.11	20.4
NiMoS_2_/Al_2_O_3 reuse SO_	31	0.17	21.4

a S_BET_: specific surface area (m^2^ g^–1^); V_pores_ (cm^3^ g^–1^): average pore volume; D_pores_ (nm): average pore diameter.

The textural characterization of the NiMoS_2_/Al_2_O_3_ catalyst after reaction revealed significant structural modifications that are consistent with deactivation phenomena commonly reported in hydrotreating processes, particularly pore blockage and the probable accumulation of carbonaceous deposits. According to the data in Table 22, the catalyst in its reduced form exhibited a high specific surface area (S_BET_ = 84 m^2^ g^–1^) and pore volume (V_pore_ = 0.27 cm^3^ g^–1^). After reuse, a drastic reduction in these parameters was observed, with S_BET_ values decreasing to 34 and 31 m^2^ g^–1^, and Vpore reduced to 0.11 and 0.17 cm^3^ g^–1^ for FO and SO feeds, respectively. This contraction suggests a significant loss of structural accessibility and the progressive coverage of active sites.^89–91^ The apparent increase in average pore diameter, from 11.8 nm to approximately 20–21 nm (Table 22), together with the reduction in surface area and pore volume, suggests significant alterations in the porous structure. Such changes are frequently associated with the accumulation of carbonaceous deposits and pore blockage in hydrotreating catalysts. According to the literature,^78^ these phenomena impose severe intraparticle diffusional limitations, restricting the mass transfer of bulky molecules. The combined textural changes observed in Table 22 suggest that polymerization reactions involving oxygenated intermediates may contribute to the degradation of textural properties and the progressive loss of catalytic activity over successive cycles.

Although direct quantification of the sulfur content by X-ray fluorescence (XRF) after reuse cycles was not reliable due to the limited sensitivity of the technique at very low sulfur concentrations, catalyst deactivation is strongly supported by the observed deterioration of textural properties (Table 22) and catalytic performance (Table 21). The loss of activity is consistent with the partial depletion and restructuring of the active NiMoS phase under sulfur-free reaction conditions. Previous studies on vegetable oil hydrotreatment have shown that oxygenated feeds promote sulfur loss from sulfided NiMo catalysts, leading to progressive deactivation when no external sulfiding agent is supplied, whereas continuous sulfur addition helps preserve catalytic activity.^53,92^ In addition, the pronounced reduction in surface area and pore volume further supports the occurrence of structural modifications during reuse. Therefore, the observed deactivation is likely the result of multiple factors acting simultaneously. However, the present experimental evidence does not allow the relative contributions of each mechanism to be distinguished quantitatively.

In addition to the probable accumulation of carbonaceous deposits and pore blockage, it should be considered that the ex situ reduction procedure adopted in this study may have allowed limited exposure of the catalyst to atmospheric oxygen during handling. Although rapid transfer to the reactor was employed to minimize this effect, partial reoxidation of the sulfided phase cannot be completely excluded and may also contribute to the progressive modification of active sites during reuse.^93^

Such morphological changes directly impacted process selectivity, as detailed in Table 18, where an increase in the formation of lighter fractions is observed at the expense of the green diesel. This shift in product distribution suggests that changes in the porous structure and in active site availability may not only contribute to catalyst deactivation but also promote secondary reactions such as cracking. Thus, the experimental results highlight a strong correlation between the preservation of the textural properties and the overall catalytic performance of the NiMoS_2_/Al_2_O_3_ system.

### 3.6 Limitations and Future Perspectives

The present study demonstrated the technical feasibility of producing renewable hydrocarbons from soybean oil and frying oil through hydrotreatment over a commercial NiMoS_2_/Al_2_O_3_ catalyst. Nevertheless, some limitations must be acknowledged. The experiments were conducted at laboratory scale, catalyst stability was evaluated over a limited number of reuse cycles, and no catalyst regeneration procedures were investigated. The absence of continuous sulfur supplementation during operation may lead to deviations in the sulfur chemical potential required to stabilize the NiMoS active phase, potentially resulting in progressive modifications of the sulfide structure during reuse.^94,95^

Future work should focus on validating the process under continuous flow (trickle-bed) operation, improving catalyst regeneration strategies, investigating sulfur management approaches, and evaluating the influence of contaminants typically present in waste frying oils on catalyst deactivation and product quality. In addition, comprehensive characterization of spent catalysts using techniques such as thermogravimetric analysis (TGA) and temperature-programmed oxidation (TPO) is recommended to quantify carbonaceous deposits and provide a more detailed understanding of catalyst deactivation mechanisms during reuse. Further characterization of fuel properties, including density, viscosity, cetane index, and cold flow behavior, is also necessary to assess the suitability of the obtained hydrocarbons as drop-in fuels.

Although a fraction of the products was distributed within the C5–C15 range, the present work was primarily focused on renewable diesel production. Therefore, the suitability of these hydrocarbons for sustainable aviation fuel (SAF) applications cannot be established based on the current results. Dedicated fuel-property characterization and compliance assessment with aviation fuel specifications will be required in future investigations.

## 4 Final Considerations

This study demonstrated the feasibility of producing renewable hydrocarbons from soybean oil and frying oil through hydrotreatment over a commercial NiMoS_2_/Al_2_O_3_ catalyst. Triacylglyceride conversions above 99% were achieved under the optimum conditions of 370 °C, 70 bar H_2_, and 800 rpm, yielding hydrocarbon selectivities above 97% and a maximum S_GD_ of 95.12%. Statistical analysis identified temperature as the most influential operational variable, followed by hydrogen pressure, whereas stirring speed showed no significant effect on process performance.

The comparable performance observed for frying oil highlights its potential as a low-cost and sustainable feedstock for renewable fuel production. Catalyst reuse experiments confirmed good stability, maintaining conversions above 93%, although progressive catalyst deactivation, probably associated with carbonaceous deposition and changes in product distribution. Overall, the results demonstrate that commercial NiMoS_2_/Al_2_O_3_ catalysts can efficiently convert waste lipids into diesel-range renewable hydrocarbons, supporting the valorization of waste cooking oils within circular bioeconomy strategies.
